# Unravelling the metabolic impact of SBS-associated microbial dysbiosis: Insights from the piglet short bowel syndrome model

**DOI:** 10.1038/srep43326

**Published:** 2017-02-23

**Authors:** Prue M. Pereira-Fantini, Sean G. Byars, James Pitt, Susan Lapthorne, Fiona Fouhy, Paul D. Cotter, Julie E. Bines

**Affiliations:** 1 Intestinal Failure and Clinical Nutrition Group, Murdoch Childrens Research Institute, Parkville, Australia; 2 Centre for Systems Genomics, School of Biosciences, The University of Melbourne, Parkville, Australia; 3 Department of Pathology, The University of Melbourne, Parkville, Australia; 4 Victorian Clinical Genetics Services, Murdoch Childrens Research Institute, Parkville, Australia; 5 Department of Paediatrics, University of Melbourne, Parkville, Australia; 6 Teagasc Food Research Centre, Moorepark, Fermoy, Ireland; 7 APC Microbiome Institute, Cork, Ireland; 8 Department of Gastroenterology and Clinical Nutrition, Royal Children’s Hospital, Parkville, Australia

## Abstract

Liver disease is a major source of morbidity and mortality in children with short bowel syndrome (SBS). SBS-associated microbial dysbiosis has recently been implicated in the development of SBS-associated liver disease (SBS-ALD), however the pathological implications of this association have not been explored. In this study high-throughput sequencing of colonic content from the well-validated piglet SBS-ALD model was examined to determine alterations in microbial communities, and concurrent metabolic alterations identified in urine samples via targeted mass spectrometry approaches (GC-MS, LC-MS, FIA-MS) further uncovered impacts of microbial disturbance on metabolic outcomes in SBS-ALD. Multi-variate analyses were performed to elucidate contributing SBS-ALD microbe and metabolite panels and to identify microbe-metabolite interactions. A unique SBS-ALD microbe panel was clearest at the genus level, with discriminating bacteria predominantly from the *Firmicutes* and *Bacteroidetes* phyla. The SBS-ALD metabolome included important alterations in the microbial metabolism of amino acids and the mitochondrial metabolism of branched chain amino acids. Correlation analysis defined microbe-metabolite clustering patterns unique to SBS-ALD and identified a metabolite panel that correlates with dysbiosis of the gut microbiome in SBS.

Short bowel syndrome (SBS) occurs as a consequence of massive small bowel resection and is clinically characterised by diarrhoea, malnutrition, metabolic derangements, prolonged hospitalisation and a severely compromised quality of life^1^. Liver disease is a major source of morbidity and mortality in children with SBS. Short bowel syndrome associated liver disease (SBS-ALD) occurs in up to 60% of children with SBS with 16.6% progressing to end stage liver disease^2^,^3^. The mechanisms underlying the development of SBS-ALD are poorly understood, however a growing body of evidence suggests SBS-associated alterations in the gut microbiome are associated with poor clinical outcomes, including parenteral nutrition dependence, bacterial translocation and bacteraemia and development of liver disease^4^,^5^,^6^. Here, we utilised a piglet SBS-ALD model to greatly improve our understanding of important bacterial changes at multiple classification levels (phylum, family, genus) due to SBS, and for the first time delineate parallel changes in metabolite profiles due to SBS-associated dysbiosis.

The gut microbiome provides a diverse range of biochemical and metabolic activities to complement the host’s physiology including the absorption, digestion, metabolism, and excretion of dietary nutrients^7^,^8^. Systems biology approaches, such as high-throughput sequencing and metabolomics, are increasingly being employed to unravel the highly complex microbe-metabolic interactions that underlie specific disease processes. High-throughput sequencing provides a culture-independent means of identifying and characterising microbial populations whilst metabolomics allows the functional status of the microbiome to be defined in biological fluids and tissues, such as urine, blood and faeces^9^. Using these technologies, specific microbial-metabolite fingerprints have been assigned to various disease states including autism^10^, infectious disease^11^,^12^, irritable bowel syndrome and Crohn’s disease^13^,^14^, thereby uncovering host-microbiome interactions linked to disease processes and improving diagnostic precision.

The piglet model of SBS-ALD exhibits morphological, microbial, metabolic and clinical features that are analogous to SBS-ALD in humans. In this model there is a decrease in colonic microbial diversity that mirrors that observed in children with SBS^5^,^6^ and a shift in the bile acid pool to a primary bile acid dominant composition^15^. These studies suggested a significant metabolic impact of SBS-associated microbial dysbiosis, but lacked the fine-scale resolution to identify key bacterial drivers of metabolic alterations in SBS-ALD, which may contribute to the development of SBS-ALD. Therefore, the aim of the current study was to use a broad, targeted mass spectrometry-based approach to investigate the impact of microbial disturbances on metabolic outcomes using urine samples obtained from SBS-ALD animals.

## Results

### The piglet model mirrors key clinical and pathological characteristics of SBS-ALD

**Figure 1:**
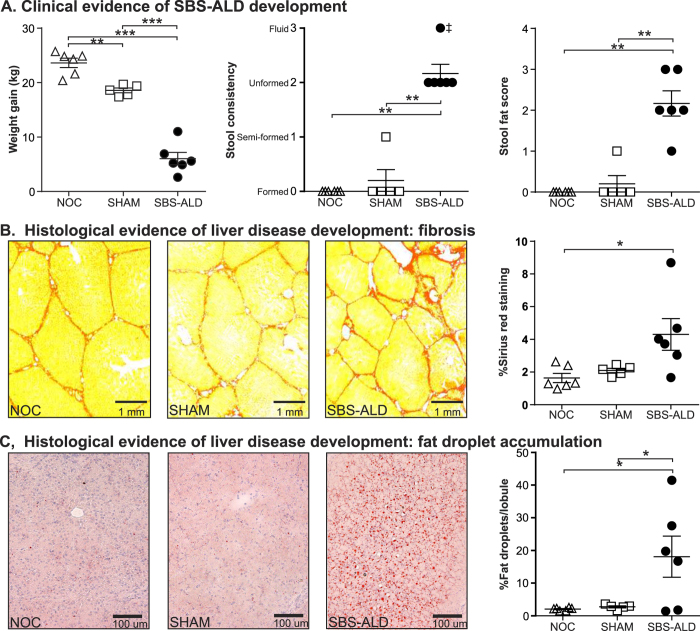
The clinical and pathological features of the piglet SBS-ALD model. (**A**) At six weeks post-surgery animals with short bowel syndrome-associated liver disease (SBS-ALD) exhibited decreased weight gain and increased stool consistency and stool fat scores indicative of persistent diarrhoea and steatorrhoea, when compared with either non-operation (NOC) or sham-operation (SHAM) control animals. Histological evidence of liver disease development in SBS-ALD animals included (**B**) increased Sirius red staining identifying increased fibrosis and (**C**) increased Oil Red O staining identifying hepatic fat droplet accumulation. Values are expressed as mean ± standard error of mean; N = 5–6/group. *P < 0.05, **P < 0.01, ***P < 0.001.

No adverse incidents resulted from either surgical procedures or the post care management of non-operation control (NOC; N = 6), sham operation control (SHAM; N = 5) or short bowel syndrome-associated liver disease (SBS-ALD; N = 6) animals. Consequently all animals were included in the subsequent study. The piglet SBS-ALD model mirrored many of the clinical and pathological features of SBS-ALD including reduced weight gain, persistent diarrhoea and steatorrhoea, and a 2.5-fold increase in hepatic Sirius red staining, indicative of fibrosis together with a 9-fold increase in hepatic fat droplet accumulation when compared against either control group (Fig. 1).

### Multi-variate analyses show clear differentiation between the SBS-ALD associated colonic microbial profile and that of the control groups

**Figure 2:**
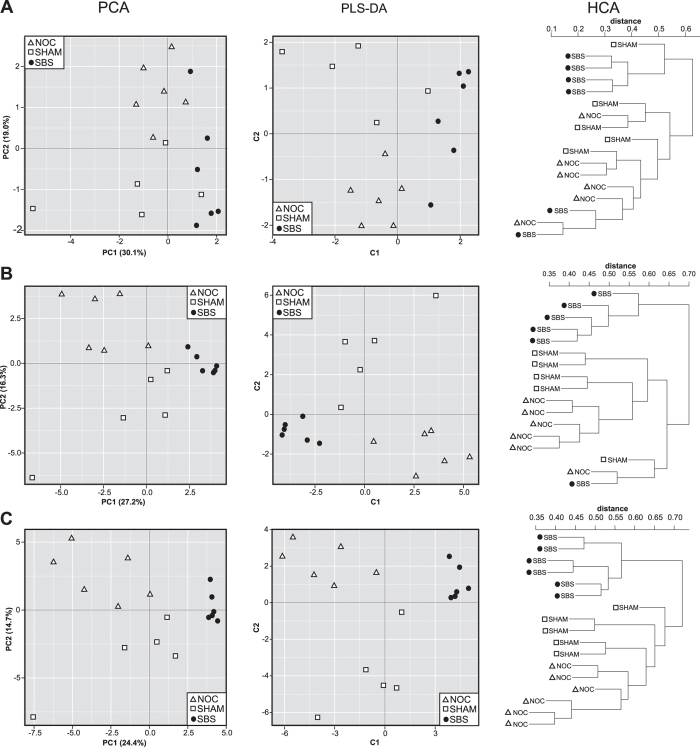
Multi-variate analysis was performed on high-throughput sequencing results obtained at the phylum (**A**), family (**B**), and genus (**C**) level using colonic content from non-operation controls (NOC), sham-operation controls (sham) or short bowel syndrome (SBS-ALD) animals.

In our initial high-throughput studies of the colonic microbiota we observed reduced microbial diversity and described changes in the relative abundance of bacteria at phylum, family and genus levels^16^. To better reflect alterations in metabolic levels, microbial alterations in the current study are reported as absolute abundance. There was no difference in the total 16 S rRNA gene copies (representative of total bacteria numbers) between the study groups (Supplementary Table 1). Extending our previous studies we performed extensive multi-variate analysis at the phylum, family and genus levels to determine if the SBS-ALD group could be distinguished from the control groups on the basis of microbial signature. PLS-DA analyses also demonstrated clear separation between SBS-ALD and both control groups across all classification levels considered (Fig. 2A–C), however this was less clear for the other methods, with PCA only showing clear separation at family and genus levels (Fig. 2B–C), and HCA only at the genus level (Fig. 2C). This reflects improved discrimination at the lowest classification level. Consequently, all further analyses were focussed at the genus level with individual animal data at each level provided in Supplementary Table 1 for completeness. Validation tests with the PLS-DA genus model showed a high goodness of fit (R^2^X(cum) = 0.633) and high predictability (Q^2^(cum) = 0.861, P < 0.05) and was considered as significantly valid since the corresponding Q^2^-intercept value was negative and the cross-validation (CV)-ANOVA P-value was lower than 0.05 (P-value = 1.101e-08). Further, CV analyses with a random forest classifier (3-fold CV repeated 3 times, mtry parameter optimized to maximize the area under the receiver characteristic curve AUCROC) that split the data into 2/3 training and 1/3 test parts showed that prediction performance of the genus model was excellent with SBS-ALD and NOC samples being 100% correctly predicted (i.e. ROC, sensitivity and specificity were all 1.0). The reason for this exceptionally high predictive ability was due to the exceptional separation of SBS-ALD from NOC at the genus level (Fig. 2C).

To help define which bacteria contributed to the separation in microbe profile between SBS-ALD and control animals, Variables Importance on PLS projections (VIP) estimates were employed. VIP is a commonly used variable selection procedure^17^,^18^,^19^ based on the Canonical Powered PLS regression. and is popular among variable selection statistics that aim to tackle the ‘large *p* variables-small *n* sample’ problem^20^,^21^ given its estimated stability and that it often outperforms other variable selection methods^22^. VIP, first published by Wold and others in 1993^22^ is calculated as a weighted sum of squares of the PLS loading weights (w) taking into account the explained variance of each PLS dimension. Given that the average of squared VIP scores is equal to 1, values greater than 1 are used as an arbitrary discriminatory cutoff point for variable/predictor selection and ranking^18^,^23^,^24^.

The highest ranked (according to VIP values) and therefore most important discriminatory bacteria that contributed to the large SBS-ALD versus control groups separation seen on component 1 (Fig. 2C and Table 1) included Proteobacteria (Genus Sutterela, 7.2-fold lower in SBS-ALD vs NOC, VIP = 2.02, Table 1), Firmicutes (Acidaminococcus, 15.3-fold higher in SBS-ALD, VIP = 1.67), Bacteriodetes (Parabacteroides, 9.1-fold lower in SBS-ALD, VIP = 1.51) then Fusobacteria (Fusobacterium, 3-fold higher in SBS-ALD, VIP = 1.44). Out of the 30 discriminatory microbes with VIP > 1 for component 1, these were predominated by Firmicutes (N = 14), Bacteriodetes (N = 5) and Proteobacteria (N = 5) (Table 1). This suggests that microbial changes in the gut associated with SBS-ALD are largely a result of shifts to many microbes within in a few main phylum, with specific microbes (within those and other phylum) playing an especially large role.

There was also separation apparent between the sham surgery group and SBS-ALD and NOC groups seen on component 2 (Fig. 2C). This may reflect changes in bacterial composition simply as a result of the surgical procedure itself. This suggests that inclusion of a surgical control in such studies is important to test and account for these effects.

### The unique SBS-ALD associated urine metabolome is dominated by alterations in the microbial metabolism of amino acids and the mitochondrial metabolism of branched chain amino acids

Microbes are a major source of metabolite production^8^. Therefore we next examined if the microbial dysbiosis observed in SBS-ALD animals was similarly mirrored by altered metabolite production. To ensure a broad detection of urinary metabolites, metabolites were detected across three platforms; gas chromatography-mass spectrometry (GC-MS), liquid chromatography-mass spectrometry (LC-MS) and flow injection analysis mass spectrometry (FIA-MS). A total of 105 metabolites from 25 classes were targeted (Supplementary Table 2). Prior to application of a Bonferroni multiple-testing threshold, 16/49 metabolites measured by GC-MS, 4/10 metabolites measured by LC-MS and 18/83 metabolites measured by FIA-MS were significantly increased (i.e. P < 0.05) in SBS-ALD animals when compared with both control groups. Removing duplications between the platforms, SBS-ALD was associated with significant increases in 30 of the 105 metabolites detected and included increases in metabolites from the following classes; benzene and substituted derivatives, fatty acyls, carboxylic acids and derivatives, diazenes, indoles and derivatives, carbohydrates and carbohydrate conjugates, and phenylpropanoic acids. This shows that there are substantial modifications to urinary metabolites in SBS-ALD animals.

**Figure 3:**
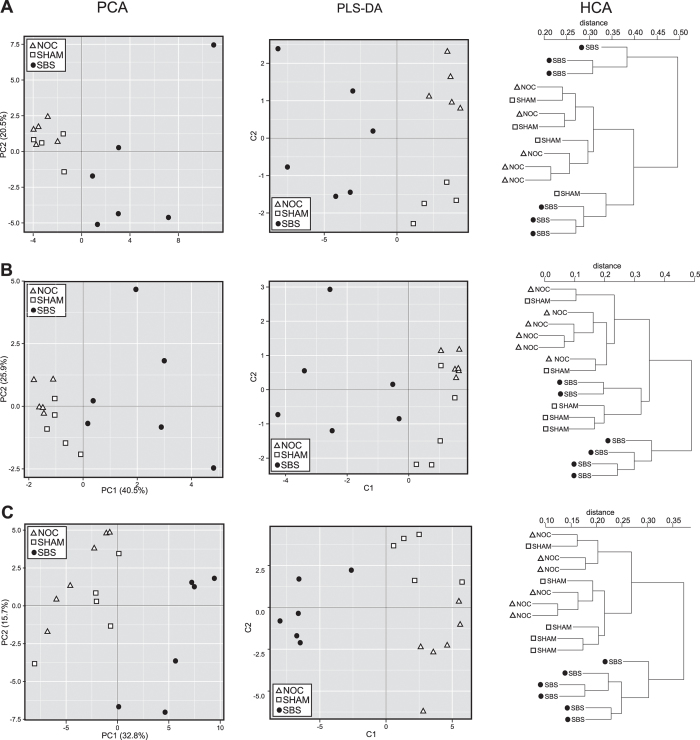
Multivariate analysis of metabolites identified in urine from non-operation control (NOC), sham-operation control (SHAM) or short bowel syndrome (SBS-ALD) via GC-MS (**A**), LC-MS (**B**) or FIA-MS (**C**).

As indicated by multi-variate analyses, the metabolite profile of SBS-ALD animals consistently separated from both control groups for both PCA and PLS-DA across all three platforms (Fig. 3A–C), however HCA analyses only showed clear separation for FIA-MS. Enhanced ability for metabolites from FIA-MS to discriminate between treatments may been due to higher final number of metabolites available for FIA-MS (N = 83) compared to GC-MS (N = 49) and LC-MS (N = 10). We therefore focused our remaining multi-variate and correlation analysis on the FIA-MS metabolite profile. The FIA-MS PLS-DA model showed a high goodness of fit (R^2^X(cum) = 0.640) and high predictability (Q^2^(cum) = 0.938, P < 0.05) and was considered as significantly valid since the corresponding Q^2^-intercept value was negative and the CV-ANOVA p-value is lower than 0.05 (p-value = 3.263e-12). CV analyses with a random forest classification also showed that prediction performance was excellent with 100% of test samples being correctly predicted (i.e. ROC, sensitivity and specificity all 1.0), which was again due to the very clear separation of these groups with the FIA-MS data (Fig. 3C).

**Figure 4:**
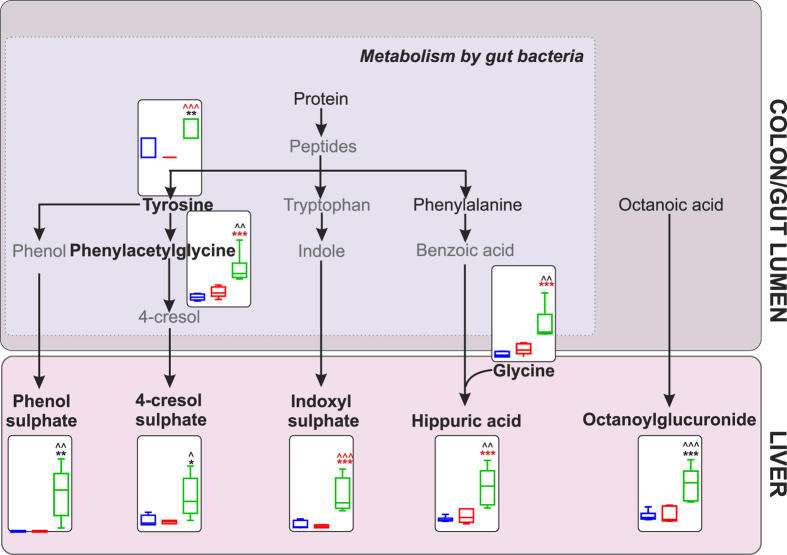
Pathway schematic detailing urine metabolite changes in SBS-ALD animals occurring as a direct or down-stream result of microbial modification. Metabolite names in black were detected in urine samples via FIA-MS. Metabolite names shown in bold represent differentially expressed metabolites and their associated abundance plot (25^th^ to 75^th^ percentile, relative to creatinine) in NOC (blue), sham (red) and SBS-ALD (green) animals, pre-multiple testing correction. *P < 0.05 NOC *vs.* SBS-ALD, **P < 0.01 NOC *vs.* SBS-ALD, ***P < 0.001 NOC *vs.* SBS-ALD, ^P < 0.05 sham *vs.* SBS-ALD, ^^P < 0.01 *vs.* SBS-ALD, ^^^P < 0.001 *vs.* SBS-ALD; black symbols representative uncorrected P value testing, red symbols represent significant metabolites following Bonferroni testing. N = 5–6/group.

We found that 45% (38/83) of FIA-MS metabolites were important for discriminating between SBS-ALD and control group separation on component 1 for PLS-DA (Supplementary Table 3). 7% (6/83) metabolites remained significantly differentially expressed between SBS-ALD and either the NOC or Sham-control group following application of a Bonferroni-based multiple testing threshold. Of these 6 metabolites, only indoxyl sulphate remained both differentially expressed against both control groups and with VIP > 1. Concurring with our observation of SBS-ALD associated microbial dysbiosis, as demonstrated in Fig. 4, 35% of the differentially expressed metabolites were either directly associated with the bacterial metabolism of protein (tyrosine and phenylacetylglycine) or were the hepatic generated downstream metabolites of the same pathway (phenol sulphate, 4-cresol sulphate, indoxyl sulphate, glycine, hippuric acid).

**Figure 5:**
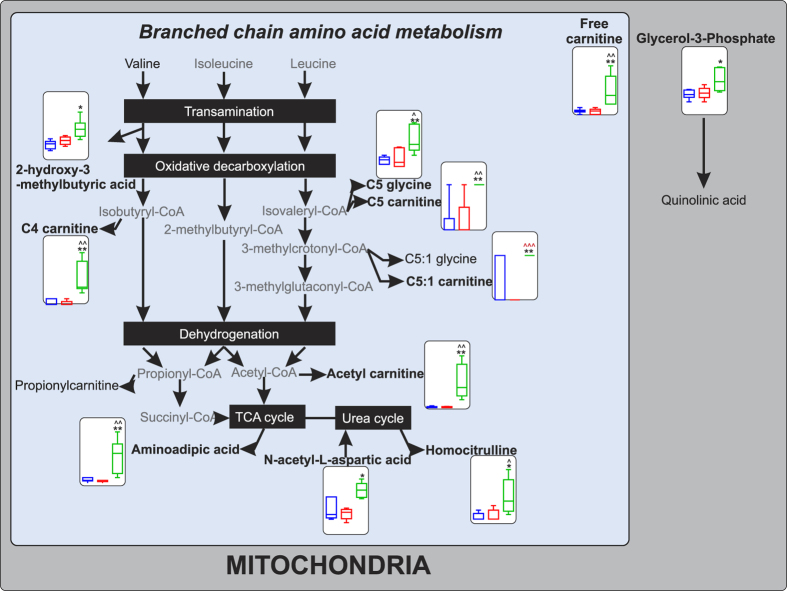
Pathway schematic detailing the mitochondrial metabolism of branched chain amino acids and the associated SBS-ALD associated urine metabolite changes. Metabolite names in black were detected in urine samples via FIA-MS. Metabolite names shown in bold represent differentially expressed metabolites and their associated abundance plot (25^th^ to 75^th^ percentile, relative to creatinine) in NOC (blue), sham (red) and SBS-ALD (green) animals, pre-multiple testing correction. *P < 0.05 NOC *vs.* SBS-ALD, **P < 0.01 NOC *vs.* SBS-ALD, ***P < 0.001 NOC *vs.* SBS-ALD, ^P < 0.05 sham *vs.* SBS-ALD, ^^P < 0.01 *vs.* SBS-ALD, ^^^P < 0.001 *vs.* SBS-ALD; black symbols representative uncorrected P value testing, red symbols represent significant metabolites following Bonferroni testing. N = 5–6/group.

As detailed in Fig. 5, the remaining metabolites, excluding octanoylglucuronide and FIGLU, were associated with the mitochondrial metabolism of branched chain amino acids.

### Correlations between microbiota and metabolome in SBS-ALD

**Figure 6:**
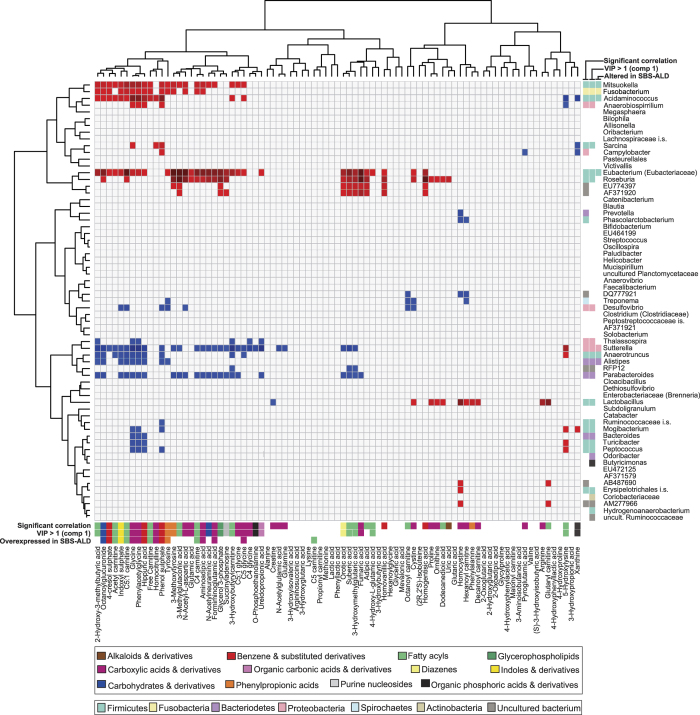
Correlation analysis of bacteria at the genus level against MSMS-derived metabolite analysis. Red squares represent positive correlations and blue squares represent negative correlations (P < 0.05). Non-significant results are shown in grey. Surrounding coloured squares act as an overview summary of the significant P values for the correlation analysis, VIP values > 1 and differential microbe/metabolite expression (uncorrected P value; P < 0.05).

Overall correlations of >0.5 or <−0.5 (*r* value) were observed between 55 metabolites and 30 microbes (Fig. 6). Amongst the metabolites, significant correlations (based on permuted p-values) were dominated by members of the carboxylic acids and derivatives and fatty acyls classes. Within the microbe panel, significant correlations were most commonly found within the Firmicutes, Proteobacteria and Bacteroidetes phyla, which were also the most important microbes for discriminating between SBS-ALD and controls in the analyses above. To delineate diagnostically relevant results we considered those microbes or metabolites which were differentially expressed in SBS-ALD animals against both control groups and which exhibited significant correlation, resulting in a 16 member metabolite panel and 7 member microbe panel. Amongst the correlations of this panel we observed a distinct clustering of positive and negative correlations such that those bacterial panel members in whom differentially expression was decreased in SBS-ALD were always negatively correlated against the metabolite panel and conversely bacteria in whom abundance was increased in SBS-ALD always exhibited a positive correlation with metabolites. These results suggest close association between microbial dysbiosis in SBS-ALD resulting in subsequent changes to metabolite profiles.

## Discussion

The gut microbiota participates in multiple host metabolic pathways and coproduces a large array of metabolites during nutrient metabolism^25^. Hence, alterations in microbial profile may have significant metabolic consequences for the host. In infants with short bowel syndrome, the development of microbial composition is interrupted, resulting in reduced microbial diversity^6^. This has been linked to the development of SBS-associated liver disease (SBS-ALD)^5^. The juvenile piglet model of SBS-ALD exhibits a high level of pathological similarity with the human condition^15^, including a reduction in microbial diversity of the microbiome^16^. By employing a multivariate analysis approach to high-throughput sequencing data obtained from the piglet SBS-ALD model, we have shown that a unique SBS-ALD microbial profile is most identifiable at the genus level, and to a lesser extent, phylum and family levels. Variable importance projection (VIP) values derived from PLS-DA analysis indicated that 46% of bacteria identified at the genus level contributed to significant discrimination between SBS-ALD and control groups, dominated by contributing members of the Firmicutes, Bacteriodetes and Proteobacteria phyla. Interestingly, separation between sham surgery and non-operation controls was consistently observed on the second component of PLS-DA analyses which supports our previous finding that minor abdominal surgery, including simple intestinal transection and re-anastmosis, can influence the microbial community^26^ and highlights the value of including both a non-operation control group and an operation control group in complex studies that may be impacted by alterations in the microbiome.

High-throughput sequencing studies have facilitated the detection of SBS-associated microbial alterations, although the potential metabolic ramifications of these compositional changes are not known. Metabolomics has emerged as a powerful exploratory tool in the identification and quantification of metabolites in a living system or biological sample. Approaches can be non-targeted such as nuclear magnetic resonance (NMR) spectroscopy or targeted via mass spectroscopy (MS) with results typically combined with multivariate data analysis. In the current study we employed a multi-platform, targeted, MS-based approach to analyse urine samples from SBS-ALD and control animals. A limitation of this approach when compared with an untargeted approach is the potential to introduce bias. However by employing three MS-based platforms (gas chromatography, liquid chromatography and flow injection analysis) we were able to detect a large panel of metabolites (105 total) from a broad range of classes (sixteen in all). Furthermore, by using metabolite analytical platforms already in routine diagnostic use, we created an opportunity to fast-track the translation of our pre-clinical results into clinical application. Differentially expressed metabolites were detected with each of the MS-platforms, with multi-variate PCA and PLS-DA analyses consistently differentiated SBS-ALD animals from both non-operation and sham-operation control groups on the first component of variation. HCA was only able to replicate this consistent separation with FIA-MS data, which is likely due to a much larger number of metabolites available for that platform and thus more variation that clustering approaches could use for discrimination. Further analysis therefore focussed on the 83 metabolites detected via FIA-MS. Variable importance in projection (VIP) estimates from PLS-DA analysis identified thirty-eight metabolites (or 45%) that contributed to the separation between SBS-ALD and control animals.

By combining the results from our multi-variate and analysis of variance statistical testing we were able to describe a SBS-ALD metabolite panel comprising of 16 metabolites that were increased in SBS-ALD when compared to both control groups and could discriminate SBS-ALD samples from those of controls. The composition of our panel suggests that SBS-ALD associated changes were restricted to alterations in metabolites resultant from (1) the metabolism of protein by bacteria or (2) the mitochondrial metabolism of branched chain amino acids and fatty acids.

Amongst the bacterially modified metabolites, SBS-ALD was associated with increased excretion of the protein-bound uraemic toxins phenol sulphate, indoxyl sulphate, 4-cresol sulphate and hippuric acid whose metabolism includes both an initial bacterial fermentation within the large intestine^27^, followed by further metabolism within the liver. In chronic liver disease patients, increased levels of these ureamic toxins are associated with liver disease prognosis in patients with cirrhosis^28^ and chronic active hepatitis^29^ suggesting that our observation of increased levels of the ureamic toxin metabolite panel may reflect liver disease progression in the SBS-ALD model.

A major observation of this study was that 13 of the differentially expressed metabolites identified were associated with mitochondrial metabolism. This is particularly relevant in the context of SBS-ALD as hepatic mitochondrial dysfunction is the result of a wide range of liver pathologies including liver cirrhosis and fatty liver and is considered a useful marker of liver disease^30^,^31^. Mirroring our own results, increased plasma levels of free carnitine and butyryl carnitine (C4 carnitine) are reported in steatosis and non-alcoholic steatohepatitis (NASH)^32^. Increased acetyl carnitine levels are reported to differentiate steatosis from NASH^33^ and correlate with the clinical stage of hepatocellular carcinoma^34^. Phenylacetylglycine is a reported biomarker of abnormal phospholipid accumulation (phopholipidosis)^35^,^36^ suggesting the current results may reflect the extensive hepatic fat accumulation observed in the piglet SBS-ALD model^15^.

The SBS-ALD metabolite panel contains both host-produced metabolites and microbe-produced metabolites that may assist in the identification of metabolic impact of SBS-ALD associated microbial disturbance. This panel included metabolites from six classes and bacteria from the Firmicutes, Fusobacteria, Proteobacteria and Bacteroidetes phyla. Very clear correlation-based clustering patterns between these bacteria and the abundance of the metabolite panel were found, for example, Mitusokella*, Fusobacterium, Acidaminococcus* and *Eubacterium* were all relatively higher in the SBS-ALD group and significantly positively correlated with the metabolite panel, whilst conversely a decreased abundance of *Suterella, Anaerotruncus* and *Alistipes* negatively correlated with the metabolite panel. Although our studies were not aimed at determining causality, these large, significant correlations suggest that the SBS-ALD associated microbial community is not only contributing to the development of the SBS-ALD metabolome but is also in turn being shaped by that same metabolome.

At present the diagnosis of SBS-ALD relies on clinical, endoscopic and histologic examination with diagnostic acuity occurring at a relative advanced stage of the disease. This study suggests that the metabolic profile in SBS is influenced by gut microbial dysbiosis and is associated with the development of SBS-ALD. We have identified a non-invasive specific SBS-ALD metabolite panel that can be measured in urine and correlates with the composition of the gut microbiome in SBS. Improved understanding of the specific characteristics of the changes in the gut microbiome and the metabolic impact of these changes may also provide specific metabolic and microbial therapeutic targets aimed at improving the clinical outcome of patients with SBS and help ameliorate the progression of SBS-ALD disease.

## Methods

### Animals and experimental design

This study was approved by the Animal Ethics Committee of the Murdoch Childrens Research Institute and performed in accordance with the guidelines of the National Health and Medical Research Committee (Australia). Weaned female 3-week-old piglets (Landrace/Large White cross; Aussie Pride Pork, Australia) were transported to The University of Melbourne Centre for Animal Biotechnology and acclimatised prior to surgery. Piglets were housed at a temperature of 22 °C with a 12 h light/dark cycle and fed a supplemented polymeric infant formula diet (Karicare De-Lact, Nutricia, Australia). The surgical procedures and peri-operative and post-operative care used in this experiment have been described previously^37^,^38^,^39^. Briefly, 4-week-old experimental piglets underwent a 75% proximal small bowel resection (SBS-ALD group; N = 6/group). Control piglets included those who underwent transection surgery (SHAM control group; N = 5/group), controlling for the effects of surgical procedures and an additional group did not undergo surgery (non-operation control group, NOC; N = 6/group). The 75% small bowel resection included the removal of the small bowel from 90 cm distal to the ligament of Treitz to 225 cm proximal to the ileocaecal valve. During the sham procedure, the intestine was transected and re-anastomosed at a site 225 cm proximal to the ileoceacal valve. Piglets received intramuscular amoxicillin (70 mg/kg; CSL Limited) 24 hours pre-surgery and on the day of surgery. Piglets received amoxicillin and oral rehydration salts (Sanofi-Aventis, Australia) for three days post-surgery in line with current clinical practice. Water and the polymeric infant formula diet were re-introduced from the third day post-operation.

### Sample collection

Animals were euthanised six weeks post-surgery. Colonic content and urine samples were obtained on the day of sacrifice and frozen at −80 °C until required. Liver samples were collected from the right medial lobe and samples were placed in 4% paraformaldehyde (Australian Biostain Pty Ltd, Traralgon, Australia) or O.C.T. compound and snap frozen in liquid nitrogen.

### Clinical assessment

Weight was recorded weekly. Stool consistency and presence of fat globules were assessed by the Royal Children’s Hospital laboratory service (Melbourne, Australia). In brief, stool was given a consistency score based on 0 = formed, 1 = semi-formed, 2 = unformed and 3 = fluid. The presence of fat globules within the stool was semi-quantitatively assessed and given a score between 0 and 3.

### Hepatic histology

Histological examination was performed on trichrome stained 4 μm formalin fixed liver sections. To measure fibrosis, Sirius Red staining was performed on 4 μm formalin-fixed liver sections and the percentage of Sirius Red staining in each liver section quantified^15^. Oil Red O staining was performed on frozen O.C.T-embedded 10 μm liver sections to visualize hepatic fat accumulation. Post staining, a minimum of 10 individual hepatic lobules were photographed per pig (Leica Microsystems, Germany) and optical density measurements were performed using the Image J software^40^.

### High-throughput sequencing

High-throughput sequencing was performed on end-point colonic samples to identify and quantify alterations in gut microbial composition. The 16 S rRNA amplicons from the colonic content were generated using a previously described approach^41^. To minimise the impact of variable starting material amounts identical amounts of the colonic samples (250 mg) was used in the DNA extraction, followed by quantifying the DNA and using identical amounts of DNA in the PCR amplification. PCR duplicates were quantified and equimolar concentrations pooled and cleaned using Agencourt AMPure kit (Beckman Coulter, USA). The V4 region of the 16 S rRNA was sequenced using a genome sequencer FLX platform (Roche Diagnostics Ltd., Switzerland). Raw sequencing reads were then quality trimmed using the RDP Pyrosequencing Pipeline applying the following criteria (i) exact matches to primer sequences and barcode tags, (ii) no ambiguous bases (Ns), and (iii) read-lengths no shorter than 150 base pairs. Trimmed FASTA sequences were then BLASTED^42^ against the SILVA (v199) database for 16 S reads^43^. Phylum, family and genus counts were extracted from MEGAN^44^ using a bit score cut-off of 86. The Qiime suite of tools^45^ was used to cluster data into operational taxonomical units (OTUs).

### Metabolomic quantification

Three different analytical platforms were used to perform targeted metabolomics analyses of urine metabolites: gas-chromatography mass spectrometry (GC-MS), liquid-chromatography mass spectrometry (LC-MS) and flow injection analysis tandem mass spectrometry (FIA-MS). Targeted metabolites were those used for diagnostic investigations of inborn errors of metabolism or metabolites previously associated with abnormal intestinal bacterial metabolism. The metabolites were identified by mass spectral and retention time matching against standards. FIA-MS analysis is unable to distinguish between some isomers and this is indicated with generic nomenclature for some metabolites e.g. C4 carnitine represents both butyryl carnitine and isobutyryl carnitine. Samples for GC-MS analysis were prepared by treatment with urease and converted to trimethylsilyl derivatives using N,O-*bis*(trimethylsilyl)trifluoroacetamide. An Agilent 5973 GC-MS system equipped with a 30 m HP-5MS column was used for the analysis. LC-MS analysis was performed on a Waters TQD system equipped with an Atlantis dC18, 2.1 × 100 mm, 3 μm column with an acetonitrile/0.1% acetic acid gradient. Electrospray ionisation was used with alternating positive and negative scans. FIA-MSMS was performed on a Waters TQD system in multiple reaction monitoring mode in positive and negative electrospray ionisation modes as previously described^46^. Urine concentration effects were corrected by diluting urines to a fixed creatinine concentration of 1 mmol/L (LC-MS) or analysing a urine volume containing a fixed amount of creatinine (200 nmoles for GC-MS and 20 nmoles for FIA-MS). Urine creatinine concentration was measured using a kinetic Jaffe reaction. Samples were mixed with an internal standard mixture and metabolite levels were expressed as a ratio of the mass spectral response (peak area of the extracted ion chromatogram of the targeted metabolite) of the metabolite relative to an internal standard using the manufacturers’ data analysis software.

### Bioinformatic and statistical analysis

#### Clinical assessment of the SBS-ALD model

Data are presented as mean values with their standard error (SEM). Within interval data sets (weight gain, Sirius red and Oil red I staining results) Shapiro-Wilk normality testing confirmed the normality of data distribution and was followed by further statistical analysis using one-way ANOVA, followed by Tukey’s post-hoc test (GraphPad Prism Software 6.0). Mann-Whitney testing was used for ordinal data sets (stool consistency and stool fat).

#### Assessing bacteria and metabolites that differed between the control and experimental groups

To assess whether control (NOC, SHAM) and experimental (SBS-ALD) groups exhibited compositional and quantitative differences in bacteria density and metabolites, we firstly used unsupervised clustering approaches including principal component analysis (PCA) and hierarchical cluster analysis (HCA; Bray-Curtis method for dissimilarity index; McQuitty method for hierarchical clustering) in R version 3.1. To further investigate which bacteria and metabolites contributed most to control and experimental group differences, we utilised partial least square discriminant analysis (PLS-DA) in the R packages ropls and MixOmics. PLS-DA models the influence of every x term (bacteria or metabolite variables) on the y variable (NOC, SHAM, SBS-ALD) and can be employed for the selection of potential biomarkers by calculating the corresponding Variables Importance on PLS projection (VIP) values. As detailed above, arbitrary VIP values of >=1 can be used to define and rank which predictor variables contribute the most to component separation found between experimental groups. The largest VIP values indicate those variables with the greatest influence of x terms on the y variable. This was assessed independently for each bacterial level (phylum, family, genus) and metabolite system (GC-MS, LC-MS, FIA-MS). Data was scaled (mean zero-centered, unit variance) prior to statistical analysis.

#### Model Validation

Final PLS-DA (i.e. genus, FIA-MS) models were validated using internal cross-validation and model quality was described with the goodness-of-fit parameters R^2^X and R^2^Y that represent the total explained variation for the X matrix and the predictive ability parameter Q^2^. High quality predictive models are indicated when the value of these parameters are greater than or equal to 0.5. Permutation tests in the PLS-DA models were performed to test for model overfitting and CV-ANOVA (analysis of variance testing of cross-validated predictive residuals) tests were performed to determine significant differences between groups in the PLS- DA models. Additionally, cross validation with a random forest classifier (R packages randomForest, pROC, caret and dplyr used) was used where the dataset with SBS-ALD and NOC samples (N = 12) was split into 2/3 training and 1/3 test parts, while keeping the proportion with and without surgery equivalent in each. The model was trained using 3-fold cross validation repeated 3 times and the mtry parameter was optimized to maximize the area under the receiver operator characteristic curve (AUCROC).

#### Determination of differential metabolite expression

To identify significant alterations in metabolite abundance normality testing was performed and upon confirmation of normal distribution of the data, parametric tests of significance were performed (one-way ANOVA followed by Tukey’s post-hoc test). P values of <0.05 were considered significant (GraphPad Prism Software 6.0).

#### Correlation between bacteria and metabolites

To test how bacterial density and metabolite levels co-vary between experimental and control, distance matrices using Manhattan distances were calculated for bacteria and metabolites separately and then Pearson correlation coefficients between both distance matrices were calculated. This was done for each mass spec system at each bacterial classification level, resulting in 3 × 3 comparisons. Significance of correlations were calculated using a Mantel test with 1000 permutations.

## Additional Information

**How to cite this article:** Pereira-Fantini, P. M. *et al*. Unravelling the metabolic impact of SBS-associated microbial dysbiosis: Insights from the piglet short bowel syndrome model. *Sci. Rep.* **7**, 43326; doi: 10.1038/srep43326 (2017).

**Publisher's note:** Springer Nature remains neutral with regard to jurisdictional claims in published maps and institutional affiliations.

## Supplementary Material

Supplementary Datasets

## Figures and Tables

**Table 1:** Complete list of genus level microbial abundance (operational taxonomic units; OTUs) and PLS-DA derived variable importance in projection (VIP) estimates obtained from high throughput sequencing of colonic content samples from non-operation control (NOC), sham-operation control (SHAM) and short bowel syndrome (SBS-ALD) animals. Mean ± SEM.

Phylum	Family	*Genus*	Bacteria abundance (OTUs)			VIP	
			NOC	SHAM	SBS-ALD	Comp 1	Comp 2
Actinobacteria	Coriobacteriaceae	*Members of the coriobacteriaceae family*	45.7 ± 13.70	16.4 ± 6.0	13.2 ± 5.6	1.15	1.04
	Bifidobacteriaceae	*Bifidobacterium*	5.30 ± 2.90	8.80 ± 7.61	5.67 ± 5.67	0.01	0.27
Bacteroidetes	Prophyromonadaceae	*Parabacteroides*	469.8 ± 147.9	264.4 ± 85.6	22.0 ± 4.99*	**1.51**	**1.10**
		***Alistipes***	**64.7 ± 20.1**	**61.0 ± 16.3**	7.33 ± 2.53*^	**1.41**	**1.10**
		*Butyricimonas*	108.8 ± 29.3	33.0 ± 8.6^#^	18.8 ± 7.1*	**1.43**	**1.25**
		*Odoribacter*	172.2 ± 66.1	37.0 ± 27.7	1.67 ± 1.67*	**1.32**	**1.11**
		*Paludibacter*	0.00 ± 0.00	5.40 ± 4.07	0.00 ± 0.00	0.14	1.03
	Bacteroidaceae	*Bacteroides*	533.7 ± 122.8	293.2 ± 48.0	160.5 ± 54.0*	**1.44**	**1.12**
	Prevotellaceae	*Prevotella*	95.3 ± 54.4	543.2 ± 173.3^#^	362.7 ± 65.6	0.75	**1.27**
Defferibacteres	Deferribacteraceae	*Mucispirillium*	1.00 ± 1.00	2.60 ± 1.60	0.00 ± 0.00	0.52	0.87
Firmicutes	Erysipelotrichales	*Turicibacter*	20.8 ± 5.3	5.00 ± 3.87^#^	0.00 ± 0.00**	**1.58**	**1.31**
		*Ersipelotrichales i.s.*	825.8 ± 616.7	246.4 ± 110.0	8.67 ± 4.88	0.85	0.68
		*Solobacterium*	20.0 ± 13.8	15.4 ± 4.4	1.17 ± 1.17	0.91	0.66
		*Catenibacterium*	14.3 ± 6.6	61.4 ± 29.0	21.2 ± 5.0	0.18	1.03
	Acidamicocccaceae	***Acidaminococcus***	**75.5 ± 48.9**	**339.6 ± 248.4**	1152 ± 257**^	**1.67**	**1.23**
		*Phascolarctobacterium*	105.5 ± 40.6	442.6 ± 147.9^#^	190.7 ± 42.9	0.19	1.27
	Veillonellaceae	***Mitsuokella***	**0.83 ± 0.83**	**41.8 ± 41.8**	744.2 ± 264.4*^	**1.54**	**1.20**
		*Allisonella*	0.00 ± 0.00	1.00 ± 1.00	2.50 ± 1.78	0.90	0.65
		*Anaerovibrio*	10.5 ± 10.5	89.8 ± 89.8	14.3 ± 2.45	0.07	0.69
		*Megasphaera*	148.0 ± 69.4	666.2 ± 290.9	727.8 ± 177.5	**1.09**	0.98
	Eubacteriaceae	***Eubacterium (Eubacteriaceae)***	**1.50 ± 1.50**	**1.20 ± 1.20**	44.67 ± 15.67*^	**1.52**	**1.21**
	Peptococcaceae	*Peptococcus*	323.2 ± 105.4	161.0 ± 58.7	1.33 ± 1.33*	**1.52**	**1.11**
	Unknown	*Mogibacterium*	21.2 ± 6.5	17.8 ± 5.3	5.83 ± 1.38	**1.23**	0.91
		*Blautia*	22.5 ± 10.1	125.2 ± 54.4^#^	14.3 ± 6.66^	**0.29**	**1.28**
		*Catabacter*	4.33 ± 2.01	1.60 ± 1.60	0.00 ± 0.00	**1.12**	0.85
		*Peptostreptococcaceae i.s.*	35.5 ± 11.6	67.0 ± 32.7	0.83 ± 0.83	0.86	**1.10**
	Ruminococcaceae	***Anaerotruncus***	**117.8 ± 28.3**	**127.2 ± 35.5**	11.0 ± 5.00*^	**1.47**	**1.23**
		*Hydrogenoanaerobacterium*	4.50 ± 2.03	0.00 ± 0.00	0.00 ± 0.00	**1.17**	**1.14**
		*Ruminococcaceae i.s.*	191.0 ± 47.5	185.0 ± 23.7	104.2 ± 19.4	1.06	0.83
		*Subdoligranulum*	158.2 ± 85.0	208.8 ± 104.1	1.67 ± 1.05	0.92	0.88
		*Oscillospira*	2.50 ± 1.59	15.8 ± 13.9	0.00 ± 0.00	0.27	0.85
		*Faecalibacterium*	0.83 ± 0.83	8.80 ± 5.96	0.00 ± 0.00	0.26	**1.03**
	Lachnospiraceae	*Roseburia*	9.67 ± 9.67	0.00 ± 0.00	50.2 ± 23.7	**1.07**	0.96
		*Lachnospiraceae i.s.*	168.2 ± 56.4	90.4 ± 23.8	262.2 ± 141.3	0.50	0.61
		*Oribacterium*	20.8 ± 15.7	33.0 ± 16.0	35.5 ± 5.58	0.47	0.40
	Clostridiaceae	*Sarcina*	0.00 ± 0.00	0.00 ± 0.00	2.33 ± 1.50	**1.06**	0.84
		*Clostridium (Clostridaceae)*	42.2 ± 12.9	116.8 ± 62.6	1.17 ± 1.17*	0.63	**1.12**
	Lactobacillaceae	*Lactobacillus*	142.2 ± 40.1	11.4 ± 7.8^#^	45.2 ± 10.0*	1.01	**1.32**
	Streptococcaceae	*Streptococcus*	1.00 ± 1.00	6.00 ± 6.00	0.00 ± 0.00	0.24	0.74
Fusobacteria	Fusobacteriaceae	***Fusobacterium***	**352.0 ± 180.4**	**194.8 ± 119.0**	1075 ± 188*^^	**1.44**	**1.28**
Lentisphaerae	Victovallaceae	*Victivallis*	13.5 ± 5.5	10.8 ± 8.9	17.0 ± 6.9	0.26	0.29
Proteobac teria	Sutterellaceae	***Sutterella***	**116.8 ± 11.1**	**43.3 ± 5.0**^**###**^	16.3 ± 2.0***^	**2.02**	**1.64**
	Rhodospirillaceae	*Thalassospira*	70.3 ± 20.2	8.80 ± 4.33^##^	0.00 ± 0.00**	**1.53**	**1.37**
	Succinivibrionaceae	*Anaerobiospirillium*	3.67 ± 2.79	10.0 ± 8.6	36.8 ± 13.7	**1.31**	0.98
	Desulfovibrionaceae	*Bilophila*	20.0 ± 5.3	17.2 ± 2.3	40.2 ± 10.9	**1.09**	0.98
		*Desulfovibrio*	160.2 ± 19.2	243.4 ± 48.5	79.3 ± 8.6^^	**1.07**	**1.42**
	Campylobacteriaceae	*Campylobacter*	2.83 ± 2.83	2.80 ± 2.80	16.33 ± 10.53	0.86	0.69
	Helicobacteriaceae	*Pasteurellales*	6.33 ± 6.33	0.00 ± 0.00	90.2 ± 89.0	0.70	0.58
	Enterobacteriaceae	*Members of the enterobacteriaceae family*	309.8 ± 90.4	62.2 ± 23.7	125.2 ± 65.4	0.86	**1.12**
	Unknown	*Helicobacter*	12.0 ± 4.3	9.60 ± 8.41	7.17 ± 4.34	0.38	0.28
Spirochaetes	Spirochaetaceae	*Treponema*	6.83 ± 6.83	37.2 ± 19.2	0.00 ± 0.00	0.41	**1.15**
Synergistetes	Synergistaceae	*Dethiosulfovibrio*	171.0 ± 64.3	44.4 ± 19.1	133.3 ± 64.2	0.16	0.81
		*Cloacibacillus*	39.0 ± 13.5	2.20 ± 1.36	23.5 ± 12.8	0.38	**1.04**
Uncultured bacterium		*AM277966*	15.3 ± 6.0	0.00 ± 0.00^#^	0.00 ± 0.00*	**1.27**	**1.24**
		*AF371579*	3.33 ± 1.59	1.00 ± 1.00	0.00 ± 0.00	**1.13**	0.89
				*RFP12*	6.67 ± 2.22	8.40 ± 4.57	0.00 ± 0.00	**1.05**	0.96
				*AB487690*	1.83 ± 1.17	0.00 ± 0.00	0.00 ± 0.00	0.92	0.90
				*AF371920*	5.83 ± 2.12	23.0 ± 16.4	45.0 ± 28.7	0.84	0.61
				*AF371921*	88.7 ± 20.7	203.6 ± 113.5	1.17 ± 1.17	0.71	**1.07**
				*EU774397*	2.83 ± 2.93	2.20 ± 1.36	9.00 ± 6.53	0.64	0.54
				*EU464199*	1.33 ± 1.33	1.60 ± 1.60	0.00 ± 0.00	0.55	0.49
				*EU472125*	1.33 ± 1.33	2.40 ± 2.40	0.00 ± 0.00	0.46	0.58
				*DQ777921*	2.00 ± 2.00	36.0 ± 26.7	0.00 ± 0.00	0.25	1.18
				*Uncultured Ruminococcaceae*	101.7 ± 62.8	53.8 ± 26.1	0.00 ± 0.00	**1.00**	0.73
				*Uncultured Planctomycetaceae*	5.67 ± 3.48	31.8 ± 18.2	0.00 ± 0.00	0.39	1.14

*p < 0.05 NOC *versus* SBS-ALD, **p < 0.01 NOC *versus* SBS-ALD, ***p < 0.001 NOC *versus* SBS-ALD. ^p < 0.05 SHAM *versus* SBS-ALD, ^^p < 0.01 SHAM *versus* SBS-ALD, ^^^p < 0.001 SHAM *versus* SBS-ALD. ^#^P < 0.05 NOC *versus* SHAM, ^##^P < 0.05 NOC *versus* SHAM, ^###^P < 0.05 NOC *versus* SHAM where black text represents significant uncorrected P values. No P values were significant when adjusted for multiple testing (Bonferroni correction threshold, P < 0.007).
