# Supplementary material for: Unravelling the metabolic impact of SBS-associated microbial dysbiosis: Insights from the piglet short bowel syndrome model
Source: Sci Rep. 2017 Feb 23;7:43326. doi: 10.1038/srep43326 (PMC5322370; doi:10.1038/srep43326)
Supplement: Supplementary Datasets [file srep43326-s1.doc]

**Supplementary Tables:**

**Unravelling the metabolic impact of SBS-associated microbial dysbiosis:**

**Insights from the piglet short bowel syndrome model**

Prue M. Pereira-Fantini^1^, Sean G. Byars^2,3^, James Pitt^4,7^, Susan Lapthorne^1^, Fiona Fouhy^5^,

Paul D. Cotter ^5,6^, Julie E. Bines^1,7,8^

^1^Intestinal Failure and Clinical Nutrition Group, Murdoch Childrens Research Institute, Parkville, Australia

^2^ Centre for Systems Genomics, School of Biosciences, The University of Melbourne, Parkville, Australia

^3^Department of Pathology, The University of Melbourne, Parkville, Australia

^4^Victorian Clinical Genetics Services, Murdoch Childrens Research Institute, Parkville, Australia

^5^Teagasc Food Research Centre, Moorepark, Fermoy, Ireland

^6^APC Microbiome Institute, Cork, Ireland

^7^Department of Paediatrics, University of Melbourne, Parkville, Australia

^8^Department of Gastroenterology and Clinical Nutrition, Royal Children’s Hospital, Parkville, Australia

**Supplementary Table 1: Abundance of specific microbiota at the phylum, family and genus level was determined in colonic samples obtained from animals assigned to either a no operation (NOC), or sham operation (SHAM) control group or a short bowel syndrome-associated liver disease (SBS-ALD) group. Values are reported as operational taxonomic units (OTUs).**

|  |  | NOC | | | | | | SHAM | | | | | SBS-ALD | | | | | |
| --- | --- | --- | --- | --- | --- | --- | --- | --- | --- | --- | --- | --- | --- | --- | --- | --- | --- | --- |
|  |  | NOC1 | NOC2 | NOC3 | NOC4 | NOC5 | NOC6 | SHAM1 | SHAM2 | SHAM3 | SHAM4 | SHAM5 | SBS-ALD1 | SBS-ALD2 | SBS-ALD3 | SBS-ALD4 | SBS-ALD5 | **SBS-ALD6** |
|  | Total reads | 14097 | 9622 | 8284 | 8080 | 4106 | 8171 | 6963 | 3460 | 10351 | 11863 | 9809 | 6507 | 6286 | 8277 | 9054 | 5670 | 8302 |
|  | No hits |  |  |  |  |  |  |  |  |  | 1 |  |  |  |  |  |  |  |
| Phylum | Actinobacteria | 67 | 32 | 113 | 43 | 52 | 13 | 5 |  | 65 | 36 | 21 | 38 | 12 | 38 | 22 |  | 16 |
|  | Bacteroidetes | 1448 | 2317 | 1558 | 2046 | 1051 | 1968 | 1411 | 904 | 2618 | 1962 | 2424 | 418 | 952 | 773 | 725 | 342 | 1036 |
|  | Defferibacteres |  |  |  |  |  | 6 | 6 |  | 7 |  |  |  |  |  |  |  |  |
|  | Firmicutes | 11605 | 5157 | 5836 | 3832 | 1624 | 5136 | 4741 | 2054 | 5820 | 9185 | 6686 | 4679 | 3109 | 5049 | 7020 | 4382 | 4885 |
|  | Fusobacteria | 64 | 617 | 56 | 1137 | 192 | 46 | 70 | 200 | 653 | 33 | 18 | 1182 | 1159 | 731 | 808 | 667 | 1905 |
|  | Lentisphaerae | 188 | 45 | 36 | 89 | 24 | 189 | 7 | 26 | 88 |  | 12 | 9 | 23 | 31 | 54 |  | 13 |
|  | Proteobacteria | 599 | 906 | 495 | 685 | 1060 | 580 | 440 | 268 | 621 | 496 | 485 | 159 | 622 | 973 | 343 | 215 | 281 |
|  | Spirochaetes |  |  |  | 42 |  |  | 47 |  | 43 |  | 104 |  |  |  |  |  |  |
|  | Synergistetes | 104 | 531 | 173 | 153 | 92 | 210 | 28 | 84 | 99 | 12 | 15 |  | 326 | 458 | 64 | 50 | 46 |
|  | 4COd-2 | 9 |  | 11 | 6 | 5 |  | 6 |  | 134 | 6 | 7 | 11 |  | 15 |  |  | 53 |
|  | Planctomycetes | 6 |  |  | 22 |  | 6 | 29 | 11 | 103 | 6 | 10 |  |  |  |  |  |  |
|  | Candidate division TM7 |  |  |  | 18 |  | 5 |  |  |  |  |  |  |  |  |  |  |  |
|  | SHA-109 |  |  |  |  |  |  | 158 | 63 | 13 | 111 |  |  |  |  |  |  |  |
|  | Verrucomicrobia |  |  |  |  |  |  |  |  | 6 |  |  |  | 9 |  |  |  |  |
|  | Deinococcus-Thermus |  |  |  |  |  |  |  |  |  |  |  |  |  |  |  |  |  |
|  | Fungi |  |  |  |  |  |  |  |  |  |  |  |  |  |  |  |  |  |
| Family | Coriobactericea | 55 | 25 | 107 | 42 | 34 | 11 | 5 |  | 26 | 31 | 20 | 37 | 11 |  | 19 |  | 12 |
|  | Bifidobacteriaceae | 8 | 6 |  |  | 34 |  |  |  | 39 | 5 |  |  |  | 34 |  |  |  |
|  | Micrococcineae |  |  |  |  |  |  |  |  |  |  |  |  |  |  |  |  |  |
|  | Actinobacteridae |  |  | 6 |  |  |  |  |  |  |  |  |  |  |  |  |  |  |
|  | Actinobacterniceae |  |  |  |  |  |  |  |  |  |  |  |  |  |  |  |  |  |
|  | Corynebacterineae |  |  |  |  |  |  |  |  |  |  |  |  |  |  |  |  |  |
|  | Bacteroidaceae | 567 | 1059 | 296 | 128 | 200 | 601 | 255 | 161 | 435 | 250 | 365 | 8 | 362 | 241 | 138 | 33 | 181 |
|  | Prevotellaceae | 7 | 351 | 17 | 128 | 55 | 14 | 244 | 233 | 343 | 795 | 1101 | 330 | 191 | 398 | 413 | 219 | 637 |
|  | Rikenellaceae | 234 | 205 | 224 | 134 | 80 | 225 | 156 | 191 | 809 | 184 | 576 | 27 | 228 | 20 | 42 | 23 | 109 |
|  | Porphyromonadaceae | 500 | 436 | 864 | 1192 | 537 | 983 | 344 | 164 | 807 | 160 | 228 | 7 | 82 | 60 | 33 | 27 | 63 |
|  | Defferibacteriaceae |  |  |  |  |  | 6 | 6 |  | 7 |  |  |  |  |  |  |  |  |
|  | Erysipelotrichales |  |  |  |  |  |  |  |  |  |  |  |  |  |  |  |  |  |
|  | Veillonellaceae | 75 | 930 | 288 | 371 | 419 | 386 | 1059 | 561 | 382 | 2942 | 3204 | 4157 | 1586 | 3711 | 6638 | 3750 | 4170 |
|  | Eubacteriaceae | 97 | 43 | 60 | 132 | 18 | 70 | 102 | 14 | 77 | 113 | 54 | 12 | 109 | 22 | 34 | 44 | 92 |
|  | Peptococcaceae | 522 | 94 | 729 | 316 | 43 | 310 | 196 | 64 | 174 | 368 | 33 |  |  |  |  | 8 |  |
|  | Ruminococcaceae | 3975 | 1885 | 3036 | 1710 | 529 | 2374 | 1805 | 636 | 2559 | 2862 | 1779 | 65 | 520 | 163 | 100 | 176 | 206 |
|  | Clostridiaceae | 97 | 57 | 45 | 22 | 7 | 35 | 285 | 42 | 253 | 5 |  | 7 |  |  | 9 | 10 |  |
|  | Lactobacillaceae | 227 | 218 | 238 | 24 | 108 | 38 | 7 | 8 |  | 42 |  | 5 | 115 | 5 | 36 | 20 | 90 |
|  | Streptococcaceae |  |  |  | 5 | 6 |  |  |  | 30 |  |  |  |  |  |  |  |  |
|  | Veillonellaceae | 75 | 930 | 288 | 371 | 419 | 386 | 1059 | 561 | 382 | 2942 | 3204 | 4157 | 1586 | 3711 | 6638 | 3750 | 4170 |
|  | Leuconostocaceae |  |  |  |  |  |  |  |  |  |  |  |  |  |  |  |  |  |
|  | Erysipeolotrichales Incertae Sedis | 3873 | 622 | 18 | 98 | 21 | 323 | 122 | 32 | 52 | 528 | 498 | 5 | 9 |  | 6 | 32 |  |
|  | Catenibacterium | 6 | 26 | 5 |  | 42 | 7 | 36 | 7 | 30 | 172 | 62 | 21 | 39 | 22 | 8 | 37 | 36 |
|  | Enterococcaceae |  |  |  |  |  |  |  |  |  |  |  |  |  |  |  |  |  |
|  | Peptostreptococcaceae (clostridiales) | 262 | 108 | 237 | 40 | 52 | 190 | 215 | 128 | 357 | 56 |  |  |  | 5 |  | 9 |  |
|  | Mollicutes | 380 |  |  | 85 |  | 94 | 11 | 5 | 29 | 56 | 32 |  |  |  |  |  |  |
|  | Erysipelotrichaceae | 17 | 15 | 36 | 7 | 38 | 12 | 5 |  | 20 |  |  |  |  |  |  |  |  |
|  | Solobacterium |  |  |  | 81 |  | 39 | 24 |  | 19 | 22 | 12 |  |  |  |  | 7 |  |
|  | Staphylococcaceae |  |  |  |  |  |  |  |  |  |  |  |  |  |  |  |  |  |
|  | Carnobacteriaceae |  |  |  |  |  |  |  |  |  |  |  |  |  |  |  |  |  |
|  | Fusobacteriaceae | 64 | 617 | 56 | 1137 | 192 | 46 | 70 | 200 | 653 | 33 | 18 | 1182 | 1159 | 731 | 808 | 667 | 1905 |
|  | Victivallaceae | 176 | 38 | 35 | 79 | 13 | 189 |  | 13 | 64 |  | 12 | 9 | 23 | 31 | 54 |  | 13 |
|  | Rhodospirillaceae | 69 | 130 | 26 | 132 | 29 | 36 | 10 |  | 27 |  | 13 |  |  |  |  |  |  |
|  | Succinivirbionaceae |  | 5 |  |  | 17 |  |  |  |  | 6 | 44 | 7 | 8 | 73 | 40 | 12 | 81 |
|  | Desulfovibrionaceae | 169 | 107 | 221 | 237 | 226 | 175 | 216 | 90 | 330 | 369 | 314 | 108 | 138 | 154 | 104 | 156 | 139 |
|  | Campylobacteraceae | 17 |  |  |  |  |  |  |  | 14 |  |  | 7 |  | 19 | 67 |  | 5 |
|  | Helicobacteraceae |  | 12 | 6 | 24 | 5 | 25 |  |  | 5 |  | 43 |  | 11 | 27 | 5 |  |  |
|  | Enterobacteriaceae | 199 | 529 | 147 | 115 | 385 | 223 | 129 | 87 | 82 | 70 | 19 | 21 | 439 | 674 | 105 | 20 | 38 |
|  | Moraxellaceae |  |  |  |  |  |  |  |  |  |  |  |  |  |  |  | 7 |  |
|  | Vibrionaceae |  |  |  |  |  |  |  |  |  |  |  |  |  |  |  | 5 |  |
|  | Pseudoalteromonadaceae |  |  |  |  |  |  |  |  |  |  |  |  |  |  |  |  |  |
|  | Alcaligenaceae | 137 | 115 | 87 | 159 | 93 | 110 | 26 | 57 | 46 | 42 | 46 | 12 | 25 | 19 | 14 | 12 | 16 |
|  | Oxalobacteraceae |  |  |  |  |  | 6 |  |  |  |  |  |  |  |  |  |  |  |
|  | Desulfuromonadales |  |  |  |  |  |  | 51 | 19 | 115 |  |  |  |  |  |  |  |  |
|  | Spirochaetaceae |  |  |  | 42 |  |  | 47 |  | 43 |  | 104 |  |  |  |  |  |  |
|  | Synergistaceae | 104 | 531 | 173 | 153 | 92 | 210 | 28 | 84 | 99 | 12 | 15 |  | 326 | 458 | 64 | 50 | 46 |
|  | Opitutae |  |  |  |  |  |  |  |  |  |  |  |  | 9 |  |  |  |  |
|  | Sharpea |  |  |  |  | 5 |  |  |  |  |  |  |  |  |  |  |  |  |
|  | Pezizomycotina |  |  |  |  |  |  |  |  |  |  |  |  |  |  |  |  |  |
|  | Lentisphaerae |  |  |  |  |  |  |  |  |  |  |  |  |  |  |  |  |  |
|  | Allobaculum |  | 6 | 6 |  |  |  |  |  |  |  |  |  |  |  |  |  |  |
|  | Planctomycetaceae | 6 |  |  | 22 |  | 6 | 29 | 11 | 103 | 6 | 10 |  |  |  |  |  |  |
|  | Thermus |  |  |  |  |  |  |  |  |  |  |  |  |  |  |  |  |  |
|  | RFP12 | 12 | 7 |  | 10 | 11 |  | 5 | 13 | 24 |  |  |  |  |  |  |  |  |
|  | EU 774397 |  |  |  |  | 17 |  |  |  |  | 6 | 5 | 6 | 41 |  |  |  | 7 |
|  | EU464199 |  |  |  |  |  | 8 |  |  | 8 |  |  |  |  |  |  |  |  |
|  | AF371513 |  |  |  |  |  |  |  |  | 8 |  |  | 53 | 14 |  |  |  |  |
|  | DQ777921 |  |  |  | 12 |  |  | 81 |  |  | 7 | 92 |  |  |  |  |  |  |
|  | EU458675 |  |  |  |  |  |  |  |  | 6 |  |  |  |  |  |  |  |  |
|  | AM930352 | 44 |  |  |  |  |  |  |  |  |  |  |  |  |  |  |  |  |
|  | EU381466 |  |  |  | 5 |  |  |  |  |  |  |  |  |  |  |  |  |  |
| Genus | Coriobacteriaceae | 55 | 25 | 107 | 42 | 34 | 11 | 5 |  | 26 | 31 | 20 | 37 | 11 |  | 19 |  | 12 |
|  | Bifidobacterium | 8 | 6 |  |  | 18 |  |  |  | 39 | 5 |  |  |  | 34 |  |  |  |
|  | Parabacteroides | 327 | 209 | 166 | 1136 | 367 | 614 | 277 | 140 | 592 | 144 | 169 |  | 35 | 23 | 19 | 24 | 31 |
|  | Alistipes | 71 | 17 | 120 | 41 | 14 | 125 | 55 | 20 | 120 | 53 | 57 |  | 12 | 15 | 8 |  | 9 |
|  | Butyricimonas | 70 | 96 | 219 | 48 | 46 | 174 | 33 | 13 | 47 | 15 | 57 |  | 43 | 27 | 13 |  | 30 |
|  | Odoribacter | 102 | 127 | 479 | 7 | 124 | 194 | 28 | 11 | 146 |  |  |  |  | 10 |  |  |  |
|  | paludibacter |  |  |  |  |  |  | 6 |  | 21 |  |  |  |  |  |  |  |  |
|  | Bacteroides | 567 | 1059 | 296 | 479 | 200 | 601 | 255 | 161 | 435 | 250 | 365 | 8 | 362 | 241 | 138 | 33 | 181 |
|  | Prevotella | 7 | 351 | 17 | 128 | 55 | 14 | 244 | 233 | 343 | 795 | 1101 | 327 | 191 | 395 | 408 | 219 | 636 |
|  | Mucispirillum |  |  |  |  |  | 6 | 6 |  | 7 |  |  |  |  |  |  |  |  |
|  | Turicibacter | 17 | 15 | 36 | 7 | 38 | 12 | 5 |  | 20 |  |  |  |  |  |  |  |  |
|  | Ersipelotrichales incertae sedis | 3873 | 622 | 18 | 98 | 21 | 323 | 122 | 32 | 52 | 528 | 498 | 5 | 9 |  | 6 | 32 |  |
|  | Solobacterium |  |  |  | 81 |  | 39 | 24 |  | 19 | 22 | 12 |  |  |  |  | 7 |  |
|  | Catenibacterium | 6 | 26 | 5 |  | 42 | 7 | 36 | 7 | 30 | 172 | 62 | 21 | 39 | 22 | 8 | 37 | 36 |
|  | Acidaminococcus | 7 | 20 | 40 |  | 72 | 314 | 277 | 102 | 6 |  | 1313 | 1081 | 235 | 1141 | 2051 | 800 | 1603 |
|  | Phascolartobacterium | 32 | 115 | 41 | 299 | 85 | 61 | 259 | 95 | 296 | 907 | 656 | 329 | 60 | 106 | 297 | 168 | 184 |
|  | Mitsuokella |  |  |  |  | 5 |  |  |  |  | 209 |  | 111 | 471 | 565 | 1125 | 1870 | 323 |
|  | Allisonella |  |  |  |  |  |  |  |  |  | 5 |  | 5 |  |  |  | 10 |  |
|  | Anaerobvibrio |  |  |  | 63 |  |  |  |  |  |  | 449 | 17 | 5 | 16 | 13 | 12 | 23 |
|  | Megasphaera | 36 | 438 | 207 |  | 200 | 7 | 520 | 294 | 77 | 1755 | 685 | 531 | 528 | 1153 | 1353 | 200 | 602 |
|  | Eubacterium (Eubacteriaceae) |  |  | 9 |  |  |  |  |  |  |  | 6 |  | 96 | 16 | 32 | 38 | 86 |
|  | Peptococcus | 516 | 90 | 723 | 288 | 42 | 280 | 190 | 61 | 157 | 365 | 32 |  |  |  |  | 8 |  |
|  | Mogibacterium | 49 | 16 | 27 | 8 | 5 | 22 | 20 |  | 13 | 28 | 28 | 8 | 10 | 6 |  | 5 | 6 |
|  | Blautia | 38 | 23 | 64 |  | 5 | 5 | 156 | 62 | 31 | 325 | 52 | 6 | 40 |  |  | 28 | 12 |
|  | Catabacter | 7 |  | 8 |  |  | 11 | 8 |  |  |  |  |  |  |  |  |  |  |
|  | Peptostreptococcaceae incertae sedis | 62 | 13 | 77 | 9 | 15 | 37 | 117 | 29 | 170 | 19 |  |  |  | 5 |  |  |  |
|  | Hydrogenoanaerobacterium | 10 |  | 9 |  |  | 8 |  |  |  |  |  |  |  |  |  |  |  |
|  | Rumminococcaceae Incertae sedis | 218 | 390 | 209 | 101 | 54 | 174 | 206 | 141 | 117 | 238 | 223 | 37 | 138 | 132 | 63 | 95 | 160 |
|  | Anaerotruncus | 231 | 156 | 90 | 71 | 35 | 124 | 94 | 47 | 244 | 170 | 81 |  | 9 |  | 13 | 11 | 33 |
|  | Subdoligranulum | 170 |  | 562 | 89 | 10 | 118 | 38 | 12 | 222 | 593 | 179 |  | 5 |  |  | 5 |  |
|  | Oscillospira | 8 |  |  |  |  | 7 | 8 |  | 71 |  |  |  |  |  |  |  |  |
|  | Faecalibacterium |  |  |  | 5 |  |  |  |  | 5 | 7 | 32 |  |  |  |  |  |  |
|  | Roseburia |  | 58 |  |  |  |  |  |  |  |  |  | 16 | 163 | 34 | 5 | 57 | 26 |
|  | Lachnospiraceae Incertae sedis | 235 | 334 | 302 | 41 | 67 | 30 | 60 | 54 | 50 | 115 | 173 | 117 | 205 | 960 | 77 | 54 | 160 |
|  | Oribacterium |  | 99 | 8 |  | 9 | 9 |  | 20 | 13 | 91 | 41 | 56 | 37 | 14 | 33 | 32 | 41 |
|  | Sarcina |  |  |  |  |  |  |  |  |  |  |  | 6 |  |  | 8 |  |  |
|  | Clostridium (Clostridiaceae) | 96 | 56 | 44 | 18 | 7 | 32 | 284 | 42 | 253 | 5 |  |  |  |  |  | 7 |  |
|  | Lactobacillus | 227 | 218 | 238 | 24 | 108 | 38 | 7 | 8 |  | 42 |  | 5 | 115 | 5 | 36 | 20 | 90 |
|  | Streptococcus |  |  |  |  | 6 |  |  |  | 30 |  |  |  |  |  |  |  |  |
|  | Fusobacterium | 64 | 617 | 56 | 1137 | 192 | 46 | 70 | 200 | 653 | 33 | 18 | 1182 | 1159 | 731 | 807 | 667 | 1905 |
|  | Victivallis | 36 | 10 |  | 17 |  | 18 |  |  | 46 |  | 8 |  | 22 | 29 | 42 |  | 9 |
|  | Sutterella | 137 | 115 | 87 | 159 | 93 | 110 | 26 | 57 | 46 | 42 | 46 | 12 | 25 | 19 | 14 | 12 | 16 |
|  | Thalassospira | 69 | 130 | 26 | 132 | 29 | 36 | 8 |  | 23 |  | 13 |  |  |  |  |  |  |
|  | Anaerobiospirrilium |  | 5 |  |  | 17 |  |  |  |  | 6 | 44 | 7 | 8 | 73 | 40 | 12 | 81 |
|  | Bilophila | 10 | 16 | 34 | 7 | 38 | 15 | 13 | 12 | 25 | 19 | 17 | 38 | 51 | 86 | 17 | 37 | 12 |
|  | Desulfovibrio | 152 | 84 | 169 | 226 | 184 | 146 | 195 | 77 | 302 | 347 | 296 | 69 | 58 | 58 | 84 | 106 | 101 |
|  | Campylobacter | 17 |  |  |  |  |  |  |  | 14 |  |  | 7 |  | 19 | 67 |  | 5 |
|  | Pateurellales |  |  |  |  | 38 |  |  |  |  |  |  |  |  | 535 | 6 |  |  |
|  | Enterobacteriaceae (Brenneria) | 199 | 529 | 147 | 115 | 646 | 223 | 129 | 87 | 78 |  | 17 | 18 | 437 | 139 | 99 | 20 | 38 |
|  | Helicobacter |  | 12 | 6 | 24 | 5 | 25 |  |  | 5 |  | 43 |  | 11 | 27 | 5 |  |  |
|  | Treponema |  |  |  | 41 |  |  | 47 |  | 35 |  | 104 |  |  |  |  |  |  |
|  | Dethiosulfovibrio | 80 | 479 | 76 | 150 | 65 | 176 | 26 | 82 | 98 | 6 | 10 |  | 270 | 388 | 49 | 47 | 46 |
|  | Cloacibacillus | 24 | 52 | 97 |  | 27 | 34 |  |  |  | 6 | 5 |  | 56 | 70 | 15 |  |  |
|  | AM277966 | 26 | 7 | 32 | 27 |  |  |  |  |  |  |  |  |  |  |  |  |  |
|  | AF371579 | 5 |  | 6 |  |  | 9 |  |  | 5 |  |  |  |  |  |  |  |  |
|  | RFP12 | 12 | 7 |  | 10 | 11 |  | 5 | 13 | 24 |  |  |  |  |  |  |  |  |
|  | AB487690 | 5 | 6 |  |  |  |  |  |  |  |  |  |  |  |  |  |  |  |
|  | AF371920 | 13 | 8 |  | 5 |  | 9 | 7 | 13 | 7 |  | 88 | 22 | 185 | 5 | 6 | 8 | 44 |
|  | AF371921 | 115 | 174 | 83 | 27 | 59 | 74 | 86 | 106 | 656 | 111 | 59 |  |  |  |  | 7 |  |
|  | EU774397 |  |  |  |  | 17 |  |  |  |  | 6 | 5 | 6 | 41 |  |  |  | 7 |
|  | EU464199 |  |  |  |  |  | 8 |  |  | 8 |  |  |  |  |  |  |  |  |
|  | EU472125 | 8 |  |  |  |  |  |  |  | 12 |  |  |  |  |  |  |  |  |
|  | DQ777921 |  |  |  | 12 |  |  | 81 |  |  | 7 | 92 |  |  |  |  |  |  |
|  | uncltured Ruminococcaceae | 394 | 20 | 19 | 154 |  | 23 | 46 | 8 | 61 | 149 | 5 |  |  |  |  |  |  |
|  | uncultured Planctomycetaceae | 6 |  |  | 22 |  | 6 | 29 | 11 | 103 | 6 | 10 |  |  |  |  |  |  |

**Supplementary Table 2**: Full list of metabolites identified in a targeted mass spectrometry analysis of urine samples from non-operation control (NOC), sham-operation control (SHAM) or short bowel syndrome (SBS-ALD) animals. Targets were identified via GC MS(⚫), LC MS (◼) or FIA MS (⧫). Where a metabolite was identified by multiple platforms the result given is in the order GC MS > LC MS > FIA MS. Colour coding indicates metabolites contained within the same superclass. *p < 0.05, **p < 0.01, ***p < 0.001 in NOC *versus* SBS-ALD. ^p < 0.05, ^^p , 0.01, ^^^p < 0.001 in SHAM *versus* SBS-AL. #p < 0.05 NOC *versus* SHAM.

|  |  |  | **Metabolite level relative to creatinine**  **(mean± SEM)** | | |
| --- | --- | --- | --- | --- | --- |
| **Class** | **Direct Parent** | **Metabolite** | **NOC** | **SHAM** | **SBS-ALD** |
| Alkaloids & derivatives | Alkaloids & derivatives | Uric acid ◼⧫ | 1099 ± 56.07 | 1273 ± 112.3 | 1227 ± 163.3 |
|  |  | Xanthine ◼⧫ | 1050 ± 109.0 | 1198 ± 134.6 | 1010 ± 196.7 |
| Benzene & substituted derivatives | Phenylsulfates | **4-cresol sulphate** ◼⧫ | **450.5 + 77.67** | **662.5 + 80.0** | **2538 + 581.2** ^^** |
|  |  | **Phenol sulphate** ◼⧫ | **85.62 + 18.23** | **225.3 + 87.7** | **1219 + 345.3** ^** |
|  | Hippuric acids | 4-Hydroxyhippuric acid ⚫ | **50.84 + 6.20** | **47.06 + 6.65** | **99.80 + 8.97** ^^** |
|  |  | Hippuric acid ⚫◼⧫ | **149.3 + 26.63** | **164.9 + 41.05** | **775.7 + 180.5*^** |
|  | Para cresols | 4-cresol ⚫ | **2.23 + 0.42** | **3.35 + 0.30** | **9.92 + 2.02** ^** |
|  | Phenols & derivatives | Phenol ⚫ | **0.62 + 0.08** | **0.55 + 0.08** | **10.92 + 2.20***^^** |
|  | Phenylacetic acid derivatives | 4-Hydroxyphenylacetic acid ⚫◼⧫ | 2.81 + 0.37 | 2.41 + 0.22 | 8.54 + 3.74 |
|  |  | Homovanillic acid ⚫⧫ | 0.79 + 0.17 | 0.67 + 0.07 | 0.92 + 0.59 |
|  |  | Homogentisic acid ⧫ | 0.01 + 0.00 | 0.01 + 0.00 | 0.01 + 0.00 |
| Fatty acyls | Acyl carntines | **Butyryl carnitine** ⧫ | **0.00 + 0.00** | **0.00 + 0.00** | **0.05 + 0.01 ** ^^** |
|  |  | **Acetyl carnitine** ⧫ | **0.02 + 0.00** | **0.02 + 0.00** | **0.46 + 0.13 ** ^^** |
|  |  | **Isovaleryl carnitine** ⧫ | **0.00 + 0.00** | **0.00 + 0.00** | **0.01 + 0.00 ** ^^** |
|  |  | **Tiglyl carnitine** ⧫ | **0.00 + 0.00** | **0.00 + 0.00** | **0.01 + 0.00 ** ^^^** |
|  |  | Propionyl carnitine ⧫ | 0.00 + 0.00 | 0.00 + 0.00 | 0.03 + 0.02 |
|  |  | Hexanoyl carnitine ⧫ | 0.00 + 0.00 | 0.00 + 0.00 | 0.00 + 0.00 |
|  |  | Octanoyl carnitine ⧫ | 0.00 + 0.00 | 0.00 + 0.00 | 0.00 + 0.00 |
|  |  | Malonyl carnitine ⧫ | 0.01 + 0.00 | 0.02 + 0.01 | 0.02 + 0.00 |
|  |  | Glutaryl carnitine ⧫ | 0.04 + 0.01 | 0.02 + 0.00 | 0.02 + 0.00 |
|  |  | 3-Hydroxybutyrylcarnitine ⧫ | 0.01 + 0.00 | 0.01 + 0.00 | 0.01 + 0.00 |
|  | Medium chain fatty acids | Adipic acid ⚫⧫ | 0.91 + 0.22 | 1.61 + 0.71 | 3.08 + 0.91 |
|  |  | Dodecanedioic acid ⚫⧫ | 1.08 + 0.13 | 0.45 +_ 0.12 | 0.88 + 0.18 |
|  |  | Suberic acid ⚫⧫ | 1.97 + 0.66 | 5.92 + 2.88 | 9.93 + 3.81 |
|  | Hydroxy fatty acids | **Free Carnitine ⧫** | **0.01 + 0.00** | **0.01 + 0.00** | **0.07 + 0.02 **^^** |
|  |  | 2-hydroxyglutaric acid | 5.77 + 2.44 | 13.71 + 14.10 | 31.93 + 24.97 |
|  |  | 3-Hydroxyisovaleric acid ⚫⧫ | 0.90 + 0.29 | 1.38 + 0.28 | 1.69 + 0.38 |
|  |  | 3-Hydroxybutyric acid ⚫⧫ | 0.54 + 0.09 | 0.94 + 0.14 | 0.89 + 0.19 |
|  |  | 3-Hydroxymethylglutaric acid ⚫⧫ | 0.58 + 0.18 | 0.68 + 0.2 | 0.95 + 0.41 |
|  |  | Mevalonic acid ⚫⧫ | 0.03 + 0.01 | 0.06 + 0.03 | 0.05 + 0.01 |
|  |  | 3-Hydroxyglutaric acid ⧫ | 0.01 + 0.00 | 0.01 + 0.00 | 0.02 + 0.00 |
|  |  | 4-Hydroxy-L-glutamic acid ⧫ | 0.00 + 0.00 | 0.01 + 0.00 | 0.01 + 0.00 |
|  |  | 5-Hydroxylysine ⧫ | 0.20 + 0.02 | 0.17 + 0.02 | 0.12 + 0.01 |
|  |  | 2-Hydroxy-3-methylbutyric acid ⚫⧫ | 0.52 + 0.10 | 0.48 + 0.15 | 0.56 + 0.34 |
|  | Glycerophosphates | **Glycerol 3-phosphate** ⚫⧫ | **11.18 + 1.36** | **11.88 + 1.77** | **37.36 + 3.90***^^^** |
|  | Bracnhed fatty acids | Ethylmalonic acid ⚫ | 0.53 + 0.18 | 0.39 + 0.18 | 0.41 + 0.22 |
|  | Methyl-branched fatty acids | 3-Methylglutaconic acid ⧫ | 0.02 + 0.00 | 0.02 + 0.00 | 0.03 + 0.00 |
| Nucleoside and nucleotide analogues | Nucleoside and nucleotide analogues | Pseudouridine ◼ | 777.5 + 77.86 | 937.3 + 147.5 | 1106 + 134.6 |
| Purine nucleosides | Purine nucleosides | Succinyladenosine ⧫ | 0.01 + 0.00 | 0.01 + 0.00 | 0.01 + 0.00 |
| Carboxylic acids & derivatives | N-acyl-aliphatic-alpha amino acids | **Phenylacetylglycine** ⚫⧫ | **134.6 + 20.32** | **244.1 + 46.75** | **862.4 + 196**^** |
|  |  | **Isovalerylglycine** ⚫⧫ | **7.32 + 1.39** | **9.31 + 2.07** | **20.50 + 3.44 **^** |
|  |  | **3-Methylcrotonyl glycine** ⧫ | **0.00 + 0.00** | **0.00 + 0.00** | **0.01 + 0.00 **^^^** |
|  |  | Suberyl glycine ⚫ | 0.81 + 0.29 | 1.81 + 0.74 | 2.40 + 0.86 |
|  |  | N-Butyryl glycine ⧫ | 0.00 + 0.00 | 0.01 + 0.00 | 0.01 + 0.00 |
|  |  | N-Acetyl-L-aspartic acid ⧫ | 0.00+ 0.00 | 0.00+ 0.00 | 0.00+ 0.00 |
|  |  | Hexanoyl glycine | 0.02 + 0.01 | 0.02 + 0.00 | 0.02 + 0.01 |
|  |  | N-Acetylglutamic acid | 0.03 + 0.00 | 0.08 + 0.03 | 0.09 + 0.01 |
|  | Alpha amino acids & derivatives | **Aminoadipic acid** ⧫ | **0.02 + 0.00** | **0.02 + 0.00** | **0.11 + 0.03 **^^** |
|  |  | **Formiminoglutamic acid** ⧫ | **0.01 + 0.00** | **0.01 + 0.00** | **0.28 + 0.11 *^** |
|  |  | **Glycine** ⧫ | **0.19 + 0.03** | **0.33 + 0.06** | **0.94 + 0.16 ***^^** |
|  |  | Creatine ◼ ⧫ | 152.5 + 37.65 | 2552 + 858.8^#^ | 1488 + 503.7 |
|  |  | Alanine ⧫ | 0.03 + 0.00 | 0.06 + 0.02 | 0.06 + 0.01 |
|  |  | (2R,2'S)-Isobuteine ⧫ | 0.01 + 0.00 | 0.01 + 0.00 | 0.01 + 0.00 |
|  |  | Pyroglutamic acid ⚫◼⧫ | 20.24 + 2.74 | 21.50 + 2.97 | 34.89 + 8.90 |
|  | Beta amino acids & derivatives | 3-Aminoisobutanoic acid ⧫ | 0.03 + 0.00 | 0.03 + 0.00 | 0.04 + 0.01 |
|  | L-alpha amino acids & derivatives | **Homocitrulline** ⧫ | **0.02 + 0.01** | **0.02 + 0.01** | **0.06 + 0.02*^** |
|  |  | Alanine ⧫ | 0.03 + 0.00 | 0.06 + 0.02 | 0.06 + 0.01 |
|  |  | Glutamic acid ⧫ | 0.02 + 0.00 | 0.02 + 0.00 | 0.03 + 0.00 |
|  |  | Glutamine ⧫ | 0.01 + 0.00 | 0.01 + 0.00 | 0.02 + 0.00 |
|  |  | Proline ⧫ | 0.06 + 0.01 | 0.04 + 0.00 | 0.07 + 0.01 |
|  |  | 4-Hydroxyproline ⧫ | 0.02 + 0.00 | 0.02 + 0.00 | 0.02 + 0.00 |
|  |  | Cystine ⧫ | 0.03 + 0.00 | 0.02 + 0.00 | 0.04 + 0.00 |
|  |  | Homocystine ⧫ | 0.01 + 0.00 | 0.00 + 0.00 | 0.00 + 0.00 |
|  |  | Arginine ⧫ | 0.03 + 0.00 | 0.02 + 0.00 | 0.02 + 0.00 |
|  |  | Lysine ⧫ | 0.13 + 0.01 | 0.18 + 0.06 | 0.18 + 0.04 |
|  | D-alpha amino acids & derivatives | Methionine ⧫ | 0.02 + 0.00 | 0.02 + 0.00 | 0.04 + 0.01 |
|  |  | Ornithine ⧫ | 0.02 + 0.00 | 0.02 + 0.00 | 0.02 + 0.00 |
|  |  | Valine ⧫ | 0.06 + 0.01 | 0.07 + 0.01 | 0.06 + 0.01 |
|  |  | Argininosuccinic acid ⧫ | 0.02 + 0.00 | 0.03 + 0.01 | 0.03 + 0.00 |
|  | Dicarboxylic acids & derivatives | Succinic acid ⚫ | 3.14 + 0.74 | 4.01 + 1.06 | 7.33 + 3.33 |
|  |  | Glutaric acid ⚫⧫ | 0.04 + 0.01 | 0.04 + 0.01 | 0.05 + 0.01 |
|  |  | Fumaric acid ⚫⧫ | 1.23 + 0.14 | 1.64 + 0.29 | 4.05 + 1.11 |
|  | Tricarboxylic acids & derivatives | **2-Methylcitric acid (peak 1)** ⚫ | **2.56 + 0.20** | **3.82 + 0.76** | **47.00 + 9.11** |
|  |  | **2-Methylcitric acid (peak 2)** ⚫ | **2.56 + 0.25** | **3.37 + 0.60** | **32.95 + 7.73** |
|  |  | Citric acid ⚫ | 99.96 + 37.36 | 76.38 + 41.99 | 109.2 + 75.63 |
|  | Peptides | Glycylproline ⧫ | 0.05 + 0.00 | 0.04 + 0.00 | 0.05 + 0.00 |
| Hydroxy acids & derivatives | Alpha hydroxy acids & derivatives | Lactic acid ⚫⧫ | 1.18 + 0.49 | 3.28 + 2.04 | 4.60 + 2.72 |
|  |  | Glycolic acid ⚫ | 2.65 + 0.17 | 1.63 + 0.52 | 1.59 + 0.66 |
|  | Beta hydroxy acids & derivatives | 3-Hydroxypropionic acid ⚫⧫ | 1.63 + 0.17 | 1.94 + 0.04 | 2.27 + 0.26 |
|  |  | (S)-3-Hydroxyisobutyric acid ⧫ | 0.02 + 0.00 | 0.02 + 0.00 | 0.01 + 0.00 |
|  | Medium chain hydroxy acids & derivatives | 3-Hydroxysebacic acid ⚫ | 0.84 + 0.32 | 2.58 + 1.23 | 4.14 + 1.71 |
| Keto acids & derivatives | Gamma-keto acids & derivatives | 2-Oxoglutaric acid ⚫⧫ | 0.68 + 0.13 | 0.64 + 0.11 | 4.88 + 3.61 |
|  | Medium chain keto acids & derivatives | 2-Oxoadipic acid ⧫ | 0.01 + 0.00 | 0.01 + 0.00 | 0.00 + 0.00 |
| Organic carbonic acids & derivatives | Ureas | Ureidopropionic acid ⧫ | 0.00 + 0.00 | 0.01 + 0.00 | 0.01 + 0.00 |
| Diazenes | Pyrimidinecarboxylic acids | Orotic acid ⚫⧫ | 3.44 + 0.64 | 2.93 + 0.28 | 4.74 + 1.45 |
|  |  | **Quinolinic acid** ⚫ | **18.03 + 1.67** | **18.73 + 2.28** | **36.21 + 2.85***^^^** |
|  | Pyrimidones | Uracil ⚫ | 0.03 + 0.01 | 0.03 + 0.01 | 0.04 + 0.01 |
| Furan | Furoic acid derivatives | 5-Hydroxymethyl-2-furancarboxylic acid ⚫ | 0.55 + 0.36 | 0.24 + 0.08 | 1.00 + 0.37 |
| Indoles & derivatives | Indoles | **Indoxyl sulphate ◼⧫** | **177.4 + 43.16** | **183.4 + 34.15** | **1408 + 290.4***^^^** |
|  | Hydroxyindoles | **5-Hydroxy-3-indoleacetic acid** ⚫ | **12.53 + 1.35** | **18.58 + 4.00** | **36.32 + 3.26***^^** |
|  | Indolyl carboxylic acids & derivatives | Indolelactic acid ⚫ | 3.82 + 0.60 | 3.60 + 0.60 | 8.48 + 2.14 |
| Alcohols & polyols | 1,2-diols | 2,3-Butanediol ⚫ | 0.01 + 0.00 | 0.01 + 0.00 | 0.04 + 0.01 |
| Carbohydrates & carbohydrate conjugates | Phenolic glycosides | **Phenol glucuronide** ⚫ | **4.55 + 2.69** | **5.23 + 2.51** | **349.8 + 90.69**^^** |
|  |  | 4-Cresol glucuronide ⚫ | 471.5 + 76.89 | 895.6 + 233.1 | 1838 + 429.7 |
|  | Sugar alcohols | **Glycerol** ⚫ | **528.1 + 16.38** | **562.8 + 23.87** | **802.4 + 79.48*^** |
|  | Neuraminic acids | N-Acetylneuraminic acid ⧫ | 0.04 + 0.00 | 0.04 + 0.01 | 0.06 + 0.01 |
|  | Sugar acids & derivatives | Glyceric acid ⚫⧫ | 10.96 + 1.72 | 12.49 + 5.97 | 20.03 + 4.44 |
|  | O-glucuronides | **Octanoylglucuronide ⧫** | **0.00 + 0.00** | **0.00 + 0.00** | **0.01 + 0.00**^^** |
| Organic phosphoric acids & derivatives | Phosphoethanolamines | O-Phosphoethanolamine **⧫** | 0.00 + 0.00 | 0.00 + 0.00 | 0.00 + 0.00 |
| Phenylpropanoic acids | Phenylpropanoic acids | **Vanillactic acid** ⚫ | **1.47 + 0.26** | **1.30 + 0.21** | **2.62 + 0.19**^^** |
|  |  | **3-(3-Hydroxyphenyl)-3-hydroxypropanoic acid** ⚫ | **0.24 + 0.03** | **0.27 + 0.06** | **0.96 + 0.23*^** |
|  |  | **Tyrosine** ⧫ | **0.02 + 0.00** | **0.02 + 0.00** | **0.03 + 0.00**^^^** |
|  |  | Phenyllactic acid ⚫⧫ | 1.8 + 0.40 | 1.65 + 0.42 | 10.31 + 7.24 |
|  |  | 4-hydroxyphenyllactic acid ⚫⧫ | 115.6 + 6.75 | 73.18 + 7.64 | 112.8 + 18.38 |
|  |  | 3-Methoxytyrosine ⧫ | 0.01 + 0.00 | 0.01 + 0.00 | 0.01 + 0.00 |
|  |  | Phenylalanine ⧫ | 0.02 + 0.01 | 0.02 + 0.00 | 0.02 + 0.00 |

**Supplementary Table 3**: FIA-MS generated list of metabolites identified in urine samples from non-operation control (NOC), sham-operation control (SHAM) or short bowel syndrome (SBS-ALD) animals. Colour coding indicates metabolites contained within the same superclass. *p < 0.05, **p < 0.01, ***p < 0.001 in NOC *versus* SBS-ALD. ^p < 0.05, ^^p , 0.01, ^^^p < 0.001 in SHAM *versus* SBS-ALD. #p < 0.05 NOC *versus* SHAM where black text represents significant uncorrected P values and red text represents significant P values adjusted for multiple testing (Bonferroni correction threshold, P < 0.0006).

|  |  |  | **Metabolite level relative to creatinine**  **(mean± SEM)** | | | **VIP** | |
| --- | --- | --- | --- | --- | --- | --- | --- |
| **Class** | **Direct Parent** | **Metabolite** | **NOC** | **SHAM** | **SBS-ALD** | **Comp1** | **Comp 2** |
| Alkaloids & derivatives | Alkaloids & derivatives | Uric acid | 0.12 + 0.01 | 0.11 + 0.02 | 0.14 + 0.12 | 0.40 | 0.53 |
|  |  | Xanthine | 0.04 + 0.01 | 0.03 + 0.01 | 0.02 + 0.00 | **1.05** | 0.80 |
| Benzene & substituted derivatives | Phenylsulfates | **4-cresol sulphate** | **0.07 + 0.02** | **0.06 + 0.01** | **0.24 + 0.06*^** | **1.33** | **1.08** |
|  |  | **Phenol sulphate** | **0.00 + 0.00** | **0.01+ 0.00** | **0.28 + 0.07**^^** | **1.53** | **1.21** |
|  | Hippuric acids | **Hippuric acid** | **0.16 + 0.02** | **0.22 + 0.05** | **0.69 + 0.12***^^** | **1.54** | **1.20** |
|  | Phenylacetic acid derivatives | 4-Hydroxyphenylacetic acid | 0.02 + 0.00 | 0.02 + 0.00 | 0.02 + 0.00 | 0.22 | 0.70 |
|  |  | Homovanillic acid | 0.00 + 0.00 | 0.00 + 0.00 | 0.01 + 0.00 | 0.52 | 0.40 |
|  |  | Homogentisic acid | 0.01 + 0.00 | 0.01 + 0.00 | 0.01 + 0.00 | 0.70 | 1.07 |
| Fatty acyls | Acyl carnitines | **C4 carnitine** | **0.00 + 0.00** | **0.00 + 0.00** | **0.05 + 0.01 ** ^^** | **1.50** | **1.19** |
|  |  | **Acetyl carnitine** | **0.02 + 0.00** | **0.02 + 0.00** | **0.46 + 0.13 ** ^^** | **1.48** | **1.18** |
|  |  | **C5 carnitine** | **0.00 + 0.00** | **0.00 + 0.00** | **0.01 + 0.00 ** ^^** | **1.20** | 0.94 |
|  |  | **C5:1 carnitine** | **0.00 + 0.00** | **0.00 + 0.00** | **0.01 + 0.00 ** ^^^** | **1.75** | **1.45** |
|  |  | Propionyl carnitine | 0.00 + 0.00 | 0.00 + 0.00 | 0.03 + 0.02 | **1.04** | 0.83 |
|  |  | Hexanoyl carnitine | 0.00 + 0.00 | 0.00 + 0.00 | 0.00 + 0.00 | 0.36 | **1.17** |
|  |  | Octanoyl carnitine | 0.00 + 0.00 | 0.00 + 0.00 | 0.01 + 0.00 | 0.91 | **1.08** |
|  |  | Malonyl carnitine | 0.01 + 0.00 | 0.02 + 0.01 | 0.02 + 0.00 | 0.14 | 0.74 |
|  |  | Glutaryl carnitine | 0.04 + 0.01 | 0.02 + 0.00^#^ | 0.02 + 0.00 | 0.53 | **1.38** |
|  |  | 3-Hydroxybutyrylcarnitine | 0.01 + 0.00 | 0.01 + 0.00 | 0.01 + 0.00 | **1.59** | **1.27** |
|  | Medium chain fatty acids | Adipic acid | 0.01 + 0.00 | 0.01 + 0.00 | 0.01 + 0.00 | 0.76 | 0.67 |
|  |  | Dodecanedioic acid | 0.00 + 0.00 | 0.00 + 0.00 | 0.00 + 0.00 | 0.12 | 0.93 |
|  |  | Suberic acid | 0.05 + 0.01 | 0.11 + 0.02 | 0.16 + 0.03* | **1.22** | **1.11** |
|  | Hydroxy fatty acids | **Free Carnitine** | **0.01 + 0.00** | **0.01 + 0.00** | **0.07 + 0.02 **^^** | **1.45** | **1.16** |
|  |  | 2-hydroxyglutaric acid | 0.03 + 0.00 | 0.06 + 0.04 | 0.07 + 0.04 | 0.42 | 0.51 |
|  |  | 3-Hydroxyisovaleric acid | 0.01 + 0.00 | 0.01 + 0.00 | 0.01 + 0.00 | 0.58 | 0.51 |
|  |  | 3-Hydroxybutyric acid | 0.04 + 0.01 | 0.04 + 0.00 | 0.05 + 0.00 | 0.76 | 0.63 |
|  |  | 3-Hydroxymethylglutaric acid | 0.01 + 0.00 | 0.01 + 0.00 | 0.02 + 0.00 | **1.01** | 0.90 |
|  |  | Mevalonic acid | 0.01 + 0.00 | 0.01 + 0.00 | 0.01 + 0.00 | 0.02 | 0.70 |
|  |  | 3-Hydroxyglutaric acid | 0.01 + 0.00 | 0.01 + 0.00 | 0.01 + 0.00 | 0.50 | 0.72 |
|  |  | 4-Hydroxy-L-glutamic acid | 0.00 + 0.00 | 0.01 + 0.00 | 0.01 + 0.00** | **1.11** | 0.87 |
|  |  | 5-Hydroxylysine | 0.20 + 0.02 | 0.17 + 0.02 | 0.12 + 0.01* | **1.31** | **1.09** |
|  |  | 2-Hydroxy-3-methylbutyric acid | 0.02 + 0.00 | 0.02 + 0.00 | 0.03 + 0.00* | **1.45** | **1.13** |
|  | Glycerophosphates | Glycerol 3-phosphate | 0.02 + 0.00 | 0.02 + 0.00 | 0.03 + 0.00* | **1.29** | **1.00** |
|  | Methyl-branched fatty acids | **3-Methylglutaconic acid** | **0.01 + 0.00** | **0.02 + 0.00** | **0.03 + 0.00** | **1.37** | **1.06** |
| Purine nucleosides | Purine nucleosides | Succinyladenosine | 0.01 + 0.00 | 0.01 + 0.00 | 0.01 + 0.00 | **1.18** | 0.92 |
| Carboxylic acids & derivatives | N-acyl-aliphatic-alpha amino acids | **Phenylacetylglycine** | **1.75 + 0.26** | **2.85 + 0.51** | **7.69 + 1.25***^^** | **1.59** | **1.23** |
|  |  | **C5 glycine** | **0.04 + 0.01** | **0.05 + 0.01** | **0.10 + 0.01**^** | **1.37** | **1.06** |
|  |  | C5:1 glycine | 0.02 + 0.00 | 0.02 + 0.01 | 0.04 + 0.00 ****^^^** | **1.29** | **1.00** |
|  |  | C4 glycine | glycerol0.00 + 0.00 | 0.01 + 0.00 | 0.01 + 0.00* | **1.16** | **1.00** |
|  |  | **N-Acetyl-L-aspartic acid** | **0.00+ 0.00** | **0.00+ 0.00** | **0.01+ 0.00*** | **1.49** | **1.31** |
|  |  | Hexanoyl glycine | 0.02 + 0.01 | 0.02 + 0.00 | 0.02 + 0.01 | 0.28 | 0.22 |
|  |  | N-Acetylglutamic acid | 0.03 + 0.00 | 0.08 + 0.03 | 0.09 + 0.01 | 0.81 | 1.31 |
|  | Alpha amino acids & derivatives | **Aminoadipic acid** | **0.02 + 0.00** | **0.02 + 0.00** | **0.11 + 0.03 **^^** | **1.43** | **1.17** |
|  |  | **Formiminoglutamic acid** | **0.01 + 0.00** | **0.01 + 0.00** | **0.28 + 0.11 *^** | **1.26** | **1.00** |
|  |  | **Glycine** | **0.19 + 0.03** | **0.33 + 0.06** | **0.94 + 0.16 ***^^** | **1.58** | **1.22** |
|  |  | Creatine | 0.05 + 0.02 | 1.04 + 0.39 | 0.82 + 0.30 | 0.57 | **1.40** |
|  |  | Alanine | 0.03 + 0.00 | 0.06 + 0.02 | 0.06 + 0.01 | 0.82 | **1.15** |
|  |  | (2R,2'S)-Isobuteine | 0.01 + 0.00 | 0.01 + 0.00 | 0.01 + 0.00 | 0.50 | 0.41 |
|  |  | Pyroglutamic acid | 0.01 + 0.00 | 0.01 + 0.00 | 0.00 + 0.00 | 0.71 | **1.03** |
|  | Beta amino acids & derivatives | 3-Aminoisobutanoic acid | 0.03 + 0.00 | 0.03 + 0.00 | 0.04 + 0.01 | 0.04 | 0.10 |
|  | L-alpha amino acids & derivatives | **Homocitrulline** | **0.02 + 0.00** | **0.02 + 0.01** | **0.06 + 0.02*^** | **1.20** | **1.00** |
|  |  | Alanine | 0.03 + 0.00 | 0.06 + 0.02 | 0.06 + 0.01 | 0.82 | **1.15** |
|  |  | Glutamic acid | 0.02 + 0.00 | 0.02 + 0.00 | 0.03 + 0.00 | **1.27** | **1.01** |
|  |  | Glutamine | 0.01 + 0.00 | 0.01 + 0.00 | 0.02 + 0.00 | 0.86 | **1.12** |
|  |  | Proline | 0.06 + 0.01 | 0.04 + 0.00 | 0.07 + 0.01 | 0.44 | **1.21** |
|  |  | 4-Hydroxyproline | 0.02 + 0.00 | 0.02 + 0.00 | 0.02 + 0.00 | 0.69 | 0.74 |
|  |  | Cystine | 0.03 + 0.00 | 0.02 + 0.00 | 0.04 + 0.00^ | 0.77 | **1.50** |
|  |  | Homocystine | 0.01 + 0.00 | 0.00 + 0.00 | 0.00 + 0.00 | 0.44 | **1.36** |
|  |  | Arginine | 0.03 + 0.00 | 0.02 + 0.00^#^ | 0.02 + 0.00 | 0.47 | **1.27** |
|  |  | Lysine | 0.13 + 0.01 | 0.18 + 0.06 | 0.18 + 0.04 | 0.34 | 0.65 |
|  | D-alpha amino acids & derivatives | Methionine | 0.02 + 0.00 | 0.02 + 0.00 | 0.04 + 0.01 | 0.72 | 0.74 |
|  |  | Ornithine | 0.02 + 0.00 | 0.02 + 0.00 | 0.02 + 0.00 | 0.27 | **1.23** |
|  |  | Valine | 0.06 + 0.01 | 0.07 + 0.01 | 0.06 + 0.01 | 0.37 | 0.82 |
|  |  | Argininosuccinic acid | 0.02 + 0.00 | 0.03 + 0.01 | 0.03 + 0.00 | 0.58 | **1.05** |
|  |  | Glutaric acid | 0.04 + 0.01 | 0.04 + 0.01 | 0.05 + 0.01 | 0.20 | 0.16 |
|  | Dicarboxylic acids & derivatives | Fumaric acid | 0.00 + 0.00 | 0.01 + 0.00 | 0.01 + 0.00 | **1.04** | 0.81 |
|  | Peptides | Glycylproline | 0.05 + 0.00 | 0.04 + 0.00 | 0.05 + 0.00 | 0.12 | 0.76 |
| Hydroxy acids & derivatives | Alpha hydroxy acids & derivatives | Lactic acid | 0.00 + 0.00 | 0.01 + 0.00 | 0.00 + 0.00 | 0.06 | 0.23 |
|  | Beta hydroxy acids & derivatives | 3-Hydroxypropionic acid | 0.01 + 0.00 | 0.01 + 0.00 | 0.01 + 0.00 | 0.80 | 0.73 |
|  |  | (S)-3-Hydroxyisobutyric acid | 0.02 + 0.00 | 0.02 + 0.00 | 0.01 + 0.00 | 0.66 | 0.51 |
| Keto acids & derivatives | Gamma-keto acids & derivatives | 2-Oxoglutaric acid | 0.00 + 0.00 | 0.00 + 0.00 | 0.00 + 0.00 | 0.40 | 0.34 |
|  | Medium chain keto acids & derivatives | 2-Oxoadipic acid | 0.01 + 0.00 | 0.01 + 0.00 | 0.01 + 0.00 | 0.37 | 0.30 |
| Organic carbonic acids & derivatives | Ureas | Ureidopropionic acid | 0.00 + 0.00 | 0.01 + 0.00 | 0.01 + 0.00** | **1.31** | **1.21** |
| Diazenes | Pyrimidinecarboxylic acids | Orotic acid | 0.01 + 0.00 | 0.02 + 0.00^#^ | 0.02 + 0.00** | **1.18** | **1.08** |
| Indoles & derivatives | Indoles | **Indoxyl sulphate** | **0.09 + 0.03** | **0.06 + 0.01** | **0.55 + 0.11***^^^** | **1.57** | **1.28** |
| Carbohydrates & carbohydrate conjugates | Neuraminic acids | N-Acetylneuraminic acid | 0.04 + 0.00 | 0.04 + 0.01 | 0.06 + 0.01* | **1.31** | **1.01** |
|  | Sugar acids & derivatives | Glyceric acid | 0.00 + 0.00 | 0.00 + 0.00 | 0.00 + 0.00 | 0.45 | 0.91 |
|  | O-glucuronides | **Octanoylglucuronide** | **0.00 + 0.00** | **0.00 + 0.00** | **0.01 + 0.00**^^** | **1.60** | **1.25** |
| Organic phosphoric acids & derivatives | Phosphoethanolamines | O-Phosphoethanolamine | 0.00 + 0.00 | 0.00 + 0.00 | 0.00 + 0.00 | **1.21** | 0.96 |
| Phenylpropanoic acids | Phenylpropanoic acids | **Tyrosine** | **0.02 + 0.00** | **0.02 + 0.00** | **0.03 + 0.00**^^^** | **1.50** | **1.33** |
|  |  | Phenyllactic acid | 0.01 + 0.01 | 0.00 + 0.00 | 0.01 + 0.01 | 0.13 | 0.66 |
|  |  | 4-hydroxyphenyllactic acid | 0.11 + 0.02 | 0.06 + 0.01^#^ | 0.07 + 0.01 | 0.68 | **1.53** |
|  |  | 3-Methoxytyrosine | 0.01 + 0.00 | 0.01 + 0.00 | 0.01 + 0.00 | **1.50** | **1.21** |
|  |  | Phenylalanine | 0.02 + 0.01 | 0.02 + 0.00 | 0.02 + 0.00 | 0.17 | **1.05** |

Supplementary Table 4: P values accompanying the FIA-MS generated list of metabolites following one-way ANOVA testing between non-operation control (NOC), sham-operation control (SHAM) and short bowel syndrome (SBS-ALD) animals. P < 0.05; values shown in black text and bolded. P < 0.0006 (Bonferroni threshold for multiple-testing) shown in red text and bolded.

|  | *P values* | | |
| --- | --- | --- | --- |
| Metabolite | NOC *vs.*  SHAM | NOC *vs.*  SBS-ALD | SHAM *vs.* SBS-ALD |
| Uric acid | 0.9482 | 0.7786 | 0.613 |
| Xanthine | 0.8912 | 0.0733 | 0.194 |
| **4-Cresol sulphate** | **0.9868** | **0.014** | **0.014** |
| **Phenol sulphate** | **0.9999** | **0.0011** | **0.0016** |
| **Hippuric acid** | **0.8881** | **0.0009** | **0.0034** |
| p-Hydroxyphenylacetic acid | 0.9944 | 0.2855 | 0.2834 |
| Homovanillic acid | 0.9761 | >0.9999 | 0.9761 |
| Homogentisic acid | >0.9999 | 0.1837 | 0.2107 |
| **C4 carnitine** | **0.9911** | **0.0025** | **0.0028** |
| **Acetyl carnitine** | **>0.9999** | **0.0027** | **0.0038** |
| **C5 carnitine** | **0.9858** | **0.0023** | **0.0045** |
| **C5:1 carnitine** | **0.2107** | **0.0058** | **0.0003** |
| Propionyl carnitine | >0.9999 | 0.0826 | 0.1001 |
| Hexanoyl carnitine | 0.7105 | >0.9999 | 0.7105 |
| Octanoyl carnitine | 0.9926 | 0.4611 | 0.5607 |
| Malonyl carnitine | 0.2107 | 0.7395 | 0.5471 |
| Glutaryl carnitine | **0.0209** | 0.1029 | 0.6092 |
| 3-Hydroxybutyrylcarnitine | No P values, all SE are zero | | |
| Adipic acid | 0.4925 | 0.2239 | 0.8729 |
| Dodecanedioic acid | 0.7676 | 0.748 | 0.3678 |
| Suberic acid | 0.2902 | 0.0115 | 0.2545 |
| **Free Carnitine** | **0.9916** | **0.0053** | **0.0059** |
| 2-hydroxyglutaric acid | 0.731 | 0.5638 | 0.9712 |
| 3-Hydroxyisovaleric acid | 0.6816 | 0.3301 | 0.841 |
| 3-Hydroxybutyric acid | 0.8 | 0.1943 | 0.5247 |
| 3-Hydroxymethylglutaric acid | 0.9168 | 0.225 | 0.439 |
| Mevalonic acid | 0.9948 | >0.9999 | 0.9948 |
| 3-Hydroxyglutaric acid | 0.9042 | 0.5152 | 0.799 |
| 4-Hydroxy-L-glutamic acid | 0.2994 | 0.0096 | 0.2153 |
| 5-Hydroxylysine | 0.4431 | 0.0114 | 0.1529 |
| 2-Hydroxy-3-methylbutyric acid | 0.7555 | 0.0112 | 0.0595 |
| 3-Methylglutaconic acid | 0.9747 | 0.0948 | 0.1637 |
| **Glycerol 3-phosphate** | **0.7203** | **0.017** | **0.096** |
| Pseudouridine | 0.6387 | 0.154 | 0.607 |
| Succinyladenosine | 0.46 | 0.1181 | 0.7 |
| **Phenylacetylglycine** | **0.6319** | **0.0004** | **0.0031** |
| **C5 glycine** | **0.8484** | **0.0068** | **0.0274** |
| **Aminoadipic acid** | **0.9862** | **0.0034** | **0.0036** |
| **C5:1 glycine** | **0.2107** | **0.0058** | **0.0003** |
| N-Acetyl-L-aspartic acid | >0.9999 | **0.0474** | **0.0595** |
| C4 glycine | 0.4009 | 0.0438 | 0.4554 |
| Hexanoyl glycine | 0.9909 | 0.8695 | 0.9336 |
| N-Acetylglutamic acid | 0.0782 | 0.0587 | 0.9991 |
| **Formiminoglutamic acid** | **0.9993** | **0.0281** | **0.0341** |
| **Glycine** | **0.6468** | **0.0004** | **0.0036** |
| Creatine | 0.0588 | 0.1291 | 0.838 |
| (2R,2'S)-Isobuteine | 0.9638 | 0.3887 | 0.2976 |
| Pyroglutamic acid | 0.9926 | 0.4611 | 0.5607 |
| Lactic acid | 0.81 | 0.88 | 0.98 |
| **Homocitrulline** | **0.9773** | **0.0161** | **0.0317** |
| Alanine | 0.1585 | 0.11 | 0.9927 |
| Glutamic acid | 0.9884 | 0.1737 | 0.1584 |
| Glutamine | 0.486 | 0.1103 | 0.6496 |
| Proline | 0.2211 | 0.957 | 0.1433 |
| 4-Hydroxyproline | 0.8761 | 0.4229 | 0.7452 |
| Cystine | 0.0983 | 0.7832 | 0.0304 |
| Homocystine | 0.0595 | 0.4062 | 0.4388 |
| Arginine | **0.022** | 0.1737 | 0.4529 |
| Lysine | 0.6236 | 0.6584 | 0.9946 |
| Methionine | 0.7763 | 0.4157 | 0.1674 |
| Ornithine | 0.6162 | 0.893 | 0.8626 |
| Valine | 0.4739 | 0.9902 | 0.4053 |
| Argininosuccinic acid | 0.2721 | 0.3843 | 0.9478 |
| 3-Aminoisobutanoic acid | 0.9953 | 0.9685 | 0.9896 |
| Glycylproline | 0.3765 | >0.9999 | 0.3765 |
| C5 dicarboxylic | 0.974 | 0.8762 | 0.9662 |
| Fumaric acid | 0.9726 | 0.2166 | 0.3392 |
| 3-Hydroxypropionic acid | 0.9906 | 0.3795 | 0.347 |
| (S)-3-Hydroxyisobutyric acid | 0.9424 | 0.5011 | 0.3556 |
| 2-Oxoglutaric acid | 0.4945 | 0.5801 | 0.9796 |
| 2-Oxoadipic acid | 0.9761 | 0.5304 | 0.6864 |
| Ureidopropionic acid | 0.0523 | 0.0038 | 0.4788 |
| Orotic acid | **0.0449** | **0.0058** | 0.654 |
| **Indoxyl sulphate** | **0.9962** | **0.0006** | **0.0006** |
| N-Acetylneuraminic acid | 0.9185 | 0.0298 | 0.0793 |
| Glyceric acid | 0.3765 | >0.9999 | 0.3765 |
| **Octanoylglucuronide** | **>0.9999** | **0.0058** | **0.0081** |
| O-Phosphoethanolamine | >0.9999 | 0.1837 | 0.2107 |
| Phenyllactic acid | 0.7059 | 0.9758 | 0.5849 |
| 4-hydroxyphenyllactic acid | 0.0436 | 0.748 | 0.9554 |
| **Tyrosine** | **0.4388** | **0.0037** | **0.0005** |
| 3-Methoxytyrosine | 0.5607 | 0.0691 | 0.4288 |
| Phenylalanine | 0.5832 | 0.9842 | 0.7602 |
